# The V‐Shaped Hamilton Receptors: A Paradigmatic Multipurpose Scaffold

**DOI:** 10.1002/open.202500120

**Published:** 2026-01-26

**Authors:** Shafieq Ahmad Wagay, Rashid Ali

**Affiliations:** ^1^ Organic and Supramolecular Functional Materials Research Laboratory Department of Chemistry Jamia Millia Islamia Okhla New Delhi 110025 India

**Keywords:** catalysis, drugs, Hamilton receptor, photoinduced electron transfer, recognition, rotaxanes

## Abstract

Sensors are devices that can detect and enumerate the physical and/or chemical aspects in real time. The generation of novel sensory materials for sensing/recognition of chemical entities are significant for protecting both the environment and humanity. This review article reveals the achievements made in the designed synthesis and molecular recognition of Hamilton‐type receptors since first report by Hamilton in 1988 to till date. This is the first elaborative manuscript in which Hamilton receptor is being exposed in detail. This manuscript is divided into three parts, in which the first portion highlights the importance and urgency of molecular recognition along with the historic background of Hamilton receptor. Whereas, the middle section discloses potential applications of Hamilton receptor in sensing and recognition of barbiturate molecules, anions, neutral molecules, drug molecules, amino acids, and racemic guest molecules. Additionally, this portion also covers the exciting applications of these receptors in the domain of rotaxanes and supramolecular catalysis. The final section highlights the future aspects of Hamilton receptor. The authors believe that this review will be useful to the inspiring researchers around the world thereby, boosting the field of receptors in the territory of supramolecular chemistry and other domains of scientific fields.

## Graphical abstract

This manuscript explores the historical context and significance of molecular recognition in relation to Hamilton‐type receptors. It concludes with a discussion on future prospects for the development of novel functionalized Hamilton‐type receptors. It is hoped that this collection will offer new insights and directions for the supramolecular chemistry community.

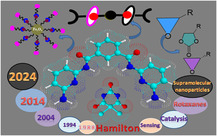

## 1 Introduction

**Figure 1:**
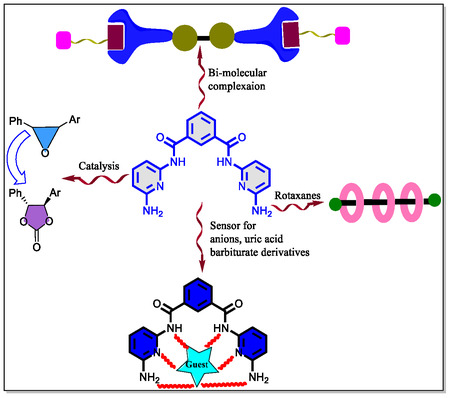
Flow chart displaying various emerging fields of Hamilton receptor.

Molecular recognition has become a promising and highly interesting research topic in the realm of supramolecular chemistry over the last few decades.^[^ ^1^, ^2^, ^3^, ^4^ ^]^ The molecular receptors have prominent features and applications in mimicking biologically active molecules as well as their utilization for drug testing and chemical production.^[^ ^5^, ^6^, ^7^, ^8^, ^9^, ^10^, ^11^, ^12^, ^13^, ^14^ ^]^ In stark contrast, multidentate metal chelons,^[^ ^15^, ^16^ ^]^ crown ethers,^[^ ^17^, ^18^ ^]^ calix[n]pyrroles,^[^ ^19^, ^20^, ^21^, ^22^, ^23^, ^24^, ^25^ ^]^ calix[n]arenes,^[^ ^26^, ^27^ ^]^ cucurbit[n]urils,^[^ ^28^, ^29^, ^30^ ^]^ cavitands,^[^ ^31^, ^32^, ^33^ ^]^ cyclodextrin,^[^ ^34^, ^35^ ^]^ and cyclophanes,^[^ ^36^, ^37^ ^]^ have also displayed a key role in the domain of molecular recognition/sensing.^[^ ^38^, ^39^, ^40^, ^41^ ^]^ Besides the above‐mentioned receptors, the molecularly imprinted polymers with their sol‐gel and anions/cations polymer pairs have also been used for designing of host cavities.^[^ ^42^ ^]^ Generally, the design of artificial receptors, which nicely mimic the naturally biological systems, is mostly based on the hydrogen bonding (H‐bonding) interactions of donor (D) and acceptor (A) systems as well as the appropriate shape of the cavity or cleft in the host molecule. To date, a myriad of receptors have been reported in the literature, whereby H‐bonding acts as the primary recognition motif like in foldamers, calix[4]pyrroles, homo‐ and hetero‐multimeric structures, 3D supramolecular host molecules, and so on.^[^ ^43^, ^44^, ^45^, ^46^, ^47^, ^48^, ^49^, ^50^ ^]^ Among these, Hamilton receptor is considered as promising moiety for molecular recognition. The Hamilton receptor (*synthetic molecular receptor*), developed by David Hamilton and his team, is known for its strong and specific hydrogen bonding interactions with certain target molecules. Compared to calixarene and cyclodextrin‐based receptors, the Hamilton receptor demonstrates superior guest selectivity, even in competitive scenarios, as it does not solely depend on size and hydrophobic interactions. The functionalization of Hamilton receptor through various chemical modification or incorporation additional recognition moieties for the enhancement of binding strength and other application is its versatility, such as attaching fluorophores for sensing applications. The inherent property of the Hamilton receptor is inspired by the development of molecules that assemble with the natural host–guest interactions, thereby, establishing it as a benchmark for effective, stable, and selective molecule recognition in supramolecular chemistry.^[^ ^51^ ^]^ The Hamilton receptor have six‐active H‐bonding sites in the two 2,6‐diamidopyridine units, which are incorporated into the molecular framework with the unique geometry, shape, and complementary cavity size. The Hamilton receptor has found diverse potential applications in sensing paradigm, recognition, and mimicking of diverse biological systems by displaying its promising role in pharmaceuticals, polymers, catalysis, and physical sciences, as well. However, from the inspection of the **Figure** **1**, it is clearly observed that the artificial receptor have been employed in diverse fields, for instance, recognition of small drug molecules and uric acid, anion sensor, rotaxanes, dendrimers, logic gates, polymeric materials, optoelectronic materials, catalysis, different stereochemistry aspects and so on. More interestingly, since from its first report in 1988 by Chang and Hamilton,^[^ ^52^ ^]^ a handful of interesting and useful literature, which incorporates its functionalization or modification of the Hamilton skeleton to generate different multifunctional receptors, have been described in detail. As far as our knowledge is concerned from the available scientific literature, this is the first review in this emerging field of research. We hope that this review article on the Hamilton‐type receptors (HR) will encourage new adventures in this emerging field.

## 2 Binding Behavior, Selectivity Trends, and Structural Influences of Hamilton Receptor

The Hamilton receptor has a preorganized host system known for its strong and predictable binding behavior toward various guest molecules through hydrogen bonding. The feature of complementary hydrogen bond donor and acceptor sites, mostly based on amide, urea, or pyridine functionalities, which result in the formation of stable 1:1 complexes with electron‐deficient guests, like barbiturates, cyanuric acid, and nucleobase analogs. It was also observed that the Hamilton‐based receptor displays high binding affinity in solvents like acetonitrile or DMSO, due to its rigid structure and directional hydrogen bonding, thereby, reducing the entropic penalties and enhancing the stability of complex. Modifications in the structural functionality toward hydrogen bonds or halogen bonding donors have extended the binding capabilities of receptor, while maintaining its preorganized nature. Furthermore, the Hamilton‐based receptor demonstrates remarkable selectivity among similar guests, driven by precise geometric complementarity and hydrogen bond. More importantly, it favors planar, tridentate guests and is sensitive to changes in guest structure. Cooperative interactions, like π–π stacking or secondary hydrogen bonds, can further enhance selectivity in specific derivatives. Structurally, the Hamilton‐based receptor benefits from its rigidity and ease of synthetic procedure that ensures optimal binding geometry. Macrocyclic, interlocked, and polymer‐bound versions of the Hamilton‐based receptor have increased its structural diversity, influencing binding dynamics and solubility. Compared to other receptor classes, such as calix[4]pyrroles, thioureas, and other hydrogen‐bonded receptors, the Hamilton‐based receptor consistently exhibits high binding constants and larger selectivity.^[^ ^51^ ^]^

## 3 Molecular Recognition and Sensing by HR

### 3.1 Barbiturate Molecules

Barbituric acid (BA) or 6‐hydroxyuracil/malonylurea, having pyrimidine heterocyclic scaffold, was first synthesized by Adolf von Baeyer in 1864 by reducing the 5,5‐dibromobarbituric acid with hydrocyanic acid. BA is vital for the synthesis of riboflavin (vitamin B2), and is also used in the production of pharmaceutical drugs, like minoxidil.^[^ ^53^ ^]^ Interestingly, its derivatives (i.e., barbiturates) are considered as emerging class of pharmaceutical species utilized for sedative,^[^ ^54^ ^]^ hypnotic,^[^ ^55^ ^]^ anticonvulsant,^[^ ^56^, ^57^ ^]^ anesthetic,^[^ ^58^ ^]^ antimicrobial,^[^ ^59^ ^]^ antitumor and anticancer activity.^[^ ^60^ ^]^ Barbital (Veronal) was utilized for the first time in 1903 for medicines and is a prototype hypnotic and sedative utilized as anxiolytic and sleeping aid, whereas phenobarbital is used for the cure of various types of epilepsy. On the contrary, merbarone acts as an antineoplastic member which display curative action against leukemia, and also shows high activity against various murine tumors.^[^ ^61^ ^]^ Besides the above medicinal applications, BA derivatives also play a key role in dye‐sensitized solar cells (DSSCs), acts as guest molecules in host–guest chemistry, and also considered as an important building block for various synthetic organic transformations.^[^ ^62^ ^]^ Therefore, due to its unique features BA molecule and other interesting properties, scientists worldwide have reported numerous versatile receptors to exhibit high selectivity as well as affinity towards barbiturate molecules.

**Figure 2:**
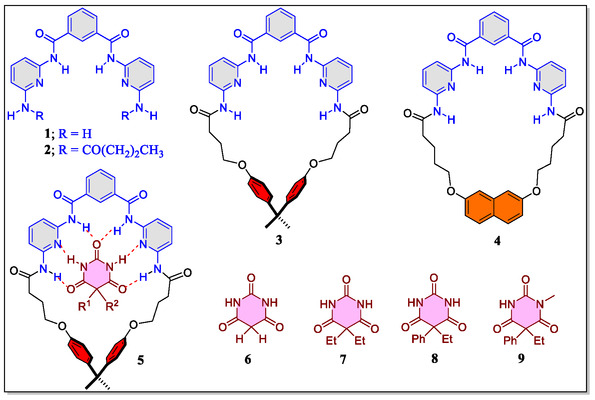
Structural representation of the Hamilton‐type receptors **1–4**, host–guest complex **5**, and some interesting barbiturate based guests molecules **6–9**.

The journey of the Hamilton receptor commenced in 1988 with the designed synthesis and studies of three different receptors (**1–3**) having six inwardly facing H‐bonding sites, which can be clearly evidenced from the host–guest complex **5** between the macrocyclic Hamilton receptor **3** and the barbiturate derivative (**Figure** **2**).^[^ ^41^ ^]^ As can be inspected from the complex **5**, the two H‐bonding acceptor–donor–acceptor (ADA) components align with the two donor–acceptor–donor (DAD) faces of barbiturate guest molecules. The macrocyclic preorganization in these types of receptors is considered very effective, thereby, displaying high guest binding affinities with varied barbiturates ranging from 10^4^ to 10^5^ M^−1^. Moreover, the HR accommodate many biologically interesting small substrates, like thymines,^[^ ^63^, ^64^, ^65^, ^66^, ^67^, ^68^ ^]^ glutarimides,^[^ ^69^, ^70^ ^]^ dipyridine‐2‐ylamines,^[^ ^71^ ^]^ uracils,^[^ ^65^ ^]^ and cyanuric acids,^[^ ^72^, ^73^, ^74^ ^]^ in their cavity or cleft through H‐bonding with strong selectivity and binding affinities. Interestingly, in this first report, the research group of Hamilton confirmed the complex formation of the receptor **2** or **3** with diverse birburates by means of ^1^H‐NMR studies (at 250 MHz instrument) in CDCl_3_ solvent through the shifting of the position of nuclear magnetic resonance (NMR) signals upfield/downfields of the proton present either in host or guest molecules. Furthermore, the authors have also confirmed the formation of complex through molecular modeling studies. The binding constants of the host–guest complexes were executed via the ^1^H‐NMR titrations utilizing either nonlinear least‐squares analysis or Foster–Fife method. From their studies, they revealed the better complementarities between the macrocyclic receptor **3** and barbiturate **7**, due to both shape as well as H‐bonding sites (association constant = 1.37 × 10^6^ M^−1^). In stark contrast, the association constant of guest molecule **7** with acyclic receptor **2** (2.08 × 10^4^ M^−1^) is almost 100‐fold less as compared with the macrocyclic receptor **3**. Whereas, the association constant of the macrocyclic receptor **3** with the barburates with mephobarbital **9** and phenobarbital **8** were estimated to be 6.80 × 10^2^ and 1.97 × 10^5^ M^−1^, respectively. Moreover, one year later to this study, they also designed a new macrocyclic receptor **4** consisting of a naphthalene moiety in its framework (Figure 2).^[^ ^52^, ^75^ ^]^ From the X‐ray analysis, the authors have noticed a tetrahydrofuran (THF) molecule sited at the central cavity of the receptor, which possess high degree of preorganization suitable for the construction of *hexa*‐hydrogen‐bonded complexes only with a slight change in conformation of the receptor. The association constants for the receptor **4** with the guests **7** and **8** is determined by means of ^1^H‐NMR titration utilizing either Foster–Fife and/or nonlinear least‐squares analysis, which were obtained to be 1.35 × 10^5^ and 2.80 × 10^5^ M^−1^, respectively. On the contrary, the Hibbs group have revealed the experimental charge density complemented by the charge density studies of macrocyclic receptor **4** and barbital **7** using quantum mechanical theoretical calculations in accordance to the Hansen–Coppens formalism.^[^ ^76^ ^]^ It was observed that the existence of barbital molecule inside the cavity stabilizes the complex due to presence of multiple H‐bonding contacts. Additionally, the authors have also found significant charge redistribution during cocrystallization, which was also confirmed through a comparison of atomic charges. Interestingly, the authors have remarked that the results in future be applied for drug designing tactics while directing toward controlling the ligand selectivity and affinity.

**Figure 3:**
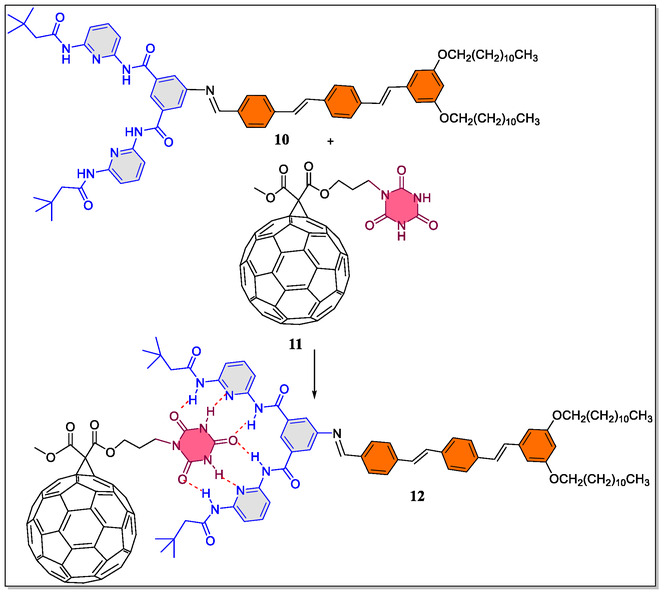
Complexation of OPV‐based Hamilton receptor **10**, with C_60_‐based cyanuric acid derivative **11**.

In contrast, Pagona et al. have successfully realized the interaction of oligophenylenevinylene (OPV)‐based Hamilton receptor with C_60_‐based cyanurate derivative by means of six complementary H‐bonding interactions (**Figure** **3**).^[^ ^77^ ^]^ The binding constant for the superstructure was examined through the ^1^H‐NMR titrations (1.95 × 10^3^ M^−1^, in toluene) besides fluorescence spectroscopic technique (1.36 × 10^5^ M^−1^, in toluene). As can be inspected from the **Table** **1**, it was observed that a comparable fluorescence quenching was noticed in more polar solvents with lower orders of magnitude as compare to the apolar solvent (toluene). On the contrary, from analysis of the Job's plots, 1:1 host–guest complex formation between **10** and **11** was observed, and also, the blue‐shift was observed from ultraviolet‐visible (UV–Vis) spectrum upon titrating **10** with **11**. Similarly, from the examination of ^1^H‐NMR studies, it was also confirmed that the regular shift of OPV‐based Hamilton and the emission of the OPV moiety **10** was quenched by addition of the fullerene (C_60_) into the solution of Hamilton receptor.

**Table 1:** Association constant and fluorescence quenching of 11 within different solvent 12.

Solvent	*K*_ass_ [M^−1^]	Fluorescence quenching [%]
Dichloromethane	4.82 × 104	30
Benzonitrile	2.82 × 104	40
Toluene	4.82 × 105	43

**Figure 4:**
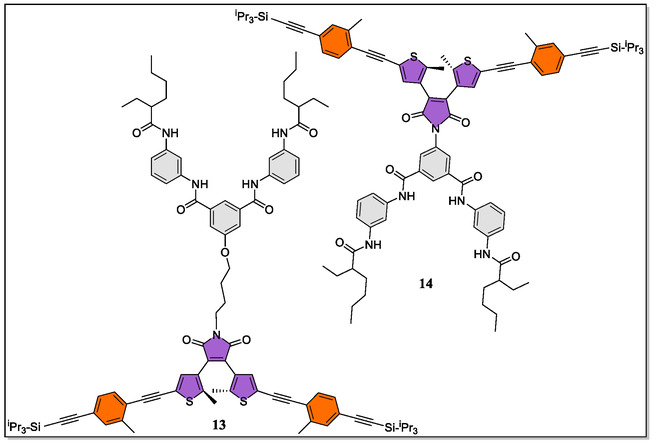
Photoswitchable dithienylethenes based Hamilton receptors **13** and **14**.

In 2018, Lüning's group have developed two potential V‐shaped Hamilton‐based photoswitchable dithienylethenes **13** and **14** by out spreading the 5,5′‐positions of dithienylethenes skeletons (**Figure** **4**).^[^ ^78^ ^]^ Interestingly, both are flexible and stiff linking was observed between the receptor and the photoswitches. Due to the extended π‐conjugation, improved conductivities in addition to the low highest occupied molecular orbital and lowest unoccupied molecular orbital (HOMO–LUMO) gap were noticed. More interestingly, these photoswitches displayed reversible switching over many cycles, and diethylbarbiturate **7** was found to bind these receptors, thereby affording valuable supramolecular complex with association constants >10^4^ M^−1^ (*regardless of open/closed forms*) as examined by means of the ^1^H‐NMR titration in CDCl_3_. Furthermore, they investigated the photoswitching properties of these vital systems (**13** and **14**) by irradiating the solutions (in CH_2_Cl_2_) with light of wavelength 311 nm (*for ring‐closing*) and 590 nm for back ring‐opening reaction. The isosbestic points observed in the UV–Vis spectra clearly revealed the generation of cyclized products. Remarkably, the authors have fruitfully followed several ring‐closing and ring‐opening cycles, and observed only very tiny fatigue. Since the HOMO–LUMO gap in these π‐extended photoswitching systems is reduced, therefore, in future, more π‐extension moieties could be installed which could facilitate electron transport properties.

**Figure 5:**
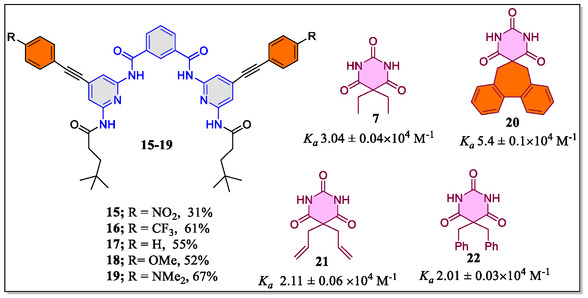
Structural representation of the Hamilton receptors **15–19** and some important barbiturates **7** and **20–22**, along with the binding affinities with the receptor **16**.

Pluth and his coworkers have reported fluorescent arylethynyl‐substituted Hamilton receptors by Sonogashira coupling reaction and studied their optoelectronic properties as well as binding studies with barburate (**Figure** **5**).^[^ ^79^ ^]^ From their studies, the authors detected absorption maxima in the range of 321–368 nm, exhibiting highest red‐shifted absorption for the compound **19**. However, in the absence of guest molecule, very weak emissions were observed for the compounds **15–19**, whereas strong emission at 451 nm was noticed for **19**. This was observed due to the presence of charge transfer (CT) (*push–pull effect*) between dimethylamino functionality (*electron donor*) and pyridine ring residue (*electron‐sink*). The CT behavior was also confirmed by virtue of the solvatochromic properties. Furthermore, the authors have pointed out that as the solvent polarity increase, the fluorescence intensity decreases, and the absorption as well as emission spectra was shifted toward longer wavelength. On the contrary, it was also reported that after the addition of barbitals, only 3–6 nm red‐shift was examined in the absorption spectra, but fluorescence spectra was much more dynamic in which electron‐withdrawing substituents displayed moderate turn‐on fluorescence, whereas electron donating substituents exhibited bimodal behavior. More importantly and surprisingly, the receptor **18** displayed strongest red‐shifted turn‐on fluorescence at 449 nm among all the tested receptors, and the receptor **19** having most powerful electron donating groups, which leads to exhibit turn‐off fluorescence (*quenching effect*). Next, the authors investigated the effect of R‐groups on the binding affinities of the barbitals molecule with the constructed novel receptor (*hosts*). As expected electron donating groups enhanced the basicity of the pyridine nitrogen (*more binding*) and electron‐withdrawing groups mitigate the basicity of the nitrogen lone pair of pyridyl group's results in lowering of H‐bonding, thereby, lowering the binding affinity. From the titration data it was also fabricated that Hammett plot (*ρ* = −0.10) display a moderate linear trend indicating that R‐groups exhibits feeble influence on the binding affinities due to large distance of pyridyl nitrogen from the R‐groups. The authors have also measured the binding of receptor **16** with a varied barbiturates using fluorescence titration, and observed no significant changes in their binding affinities.

**Figure 6:**
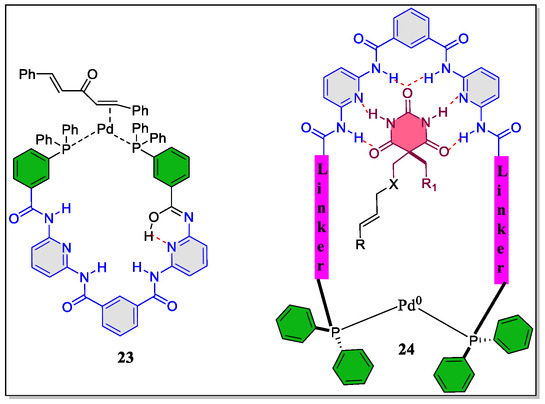
Structure of *bis*‐phosphine palladium(0) complexes of Hamilton‐type receptors.

Remarkably, the research group of Skrydstrup has synthesized various diphosphine ligands, having barbiturate‐binding ability, among them, some have displayed the ability to form 26‐membered macrocyclic *bis*‐phosphine palladium(0) complexes (**Figure** **6**).^[^ ^80^ ^]^ The aim of synthesizing palladium(0) complexes for the Heck coupling reaction was to offers the control over regioselectivity during the insertion step. In fact, their complexes were found not to catalyze the reaction with same reactivities as that of triphenylphosphine, whereas, in one case, the higher reactivity was observed by the authors.

**Figure 7:**
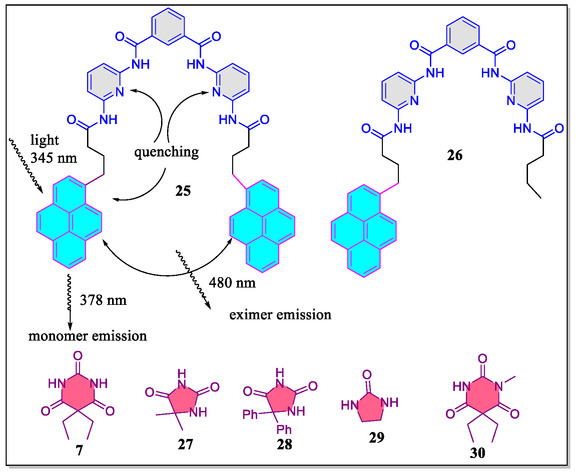
Structural view of the pyrene‐based Hamilton receptors and some guest molecules.

In a separate report, Aoki et al. have synthesized fluorescent Hamilton type receptor **25** consisting of two fluorescent pyrene moieties at the lower rim. From their studies, it was observed that the binding of guests sensitively impacts the fluorescent activities of the barbiturate‐assimilated pyrene creating ‘read‐out’ through the molecular recognition method with the usage of the fluorescence technique (**Figure** **7**).^[^ ^81^ ^]^ The authors realized that the receptor **26** gives only monomer emission, whereas, the receptor **25** provides both monomer as well as excimer emissions. Moreover, they also found that Δ*I*_m_/*I*_m_° and Δ*I*_ex_/*I*_ex_° parameters clearly indicates that the recognition vary sensitively by changing the structure of the guest molecules. In this regard, it was cleared that the barbital **7**, showed increase in Δ*I*_m_/*I*_m_° and decrease in Δ*I*_ex_/*I*_ex_°, whereas, the 5,5‐dimethylhydantoin **27**, 5,5‐diphenylhydantoin **28,** and ethyleneurea **29**, displayed increase in both Δ*I*_m_/*I*_m_° and Δ*I*_ex_/*I*_ex_° parameters (**Table** **2**). Herein, increase in the Δ*I*_m_/*I*_m_° is readily accounted by the underlying quenching efficiency of pyridine rings, whereas, the increase in Δ*I*_ex_/*I*_ex_° is attributed by the fixation mode of pyrene units (due to H‐bonding of the terminal NH). The decreasing order of various guest molecules with the receptor **25** for Δ*I*_ex_/*I*_ex_° parameter is as follow **7 **< **28 **< **29 **< **30**, it is based upon the number of H‐bonding and steric effect present in the guest molecules.

**Table 2:** Change in fluorescence induced by various guest molecules (25 °C).

Guest	**25** (CHCl_3_)		**25** (CHCl_3_–C_6_H_12_) (2:8 V/V)		**26** (CHCI_3_)	**26** (CHCl_3_–C_6_H_12_) (2:8 V/V)
	Δ*l*_m_/Δ*l*_m_°	Δ*l*_ex_/Δ*l*_ex_°	Δ*l*_m_/Δ*l*_m_°	Δ*l*_ex_/Δ*l*_ex_°	Δ*l*_m_/Δ*l*_m_°	Δ*l*_m_/Δ*l*_m_°
**7**	0.44	−0.04	1.67	−0.03	0.58	1.00
**27**	0.10	1.15	0.32	0.65	–	0.54
**28**	0.99	0.49	–	–	–	–
**29**	0.12	2.48	0.10	1.23	1.37	1.47
**30**	b	b	0.87	0.09	–	–

**Figure 8:**
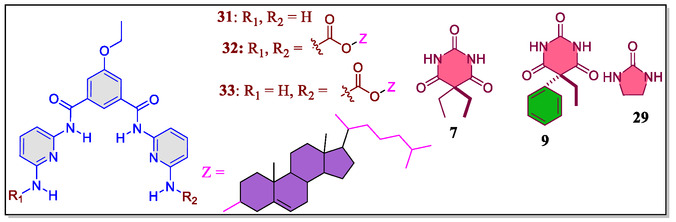
Chemical structure of the Hamilton‐type receptors (**31–33**) and guest molecules.

In a separate work, Chambers and coworkers have reported a HR containing one/two cholesteryl subunits displaying binding with barbiturates as well as changes in optical properties of cholesteric liquid crystal display (LCDs).^[^ ^82^ ^]^ In this study, the host–guest molecular recognition of barbiturates/substituted urea induced the visible changes in color of LCDs that contains *bis*(cholesteryl)‐substituted host **32** (**Figure** **8**). Moreover, it was observed that the color change of LCDs depends upon the number of equivalents of guest molecules in mesogen blend. Furthermore, it was revealed that the LCDs organized with lower amounts (2.0 mol%) of mesogen/receptor acts as the selective sensors for barbiturates. Whereas, the LCDs containing 5.4 mol% mesogen/receptor acts as a sensitive sensors in visible changes with small quantity of the guests. However, the LCDs with 5.4 mol% of **32** were not found capable to differentiate between the target analytes and feebly binding surrogates. In general, the complementary host/guest interaction of H‐bonding analytes between *bis*(cholesteryl) receptor **32** and cholesteric LCD display changes up to +70 nm, as confirmed through the naked‐eye, showing the color change from blue‐to‐orange.

### 3.2 The Uric Acid

**Figure 9:**
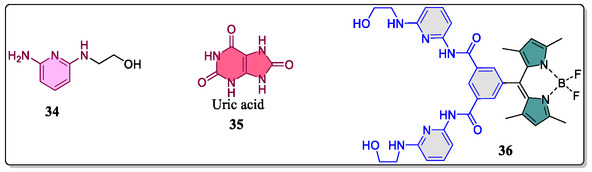
Chemical structure of BOlDIPY‐based fluorogenic probe (**36)** and guest uric acid (**35**).

Uric acid is considered as chief metabolized result of purine base in nucleic acids (*guanine and adenine*). Its normal concentration in human urine (2 mM), and in blood or serum (120–380 μM), is maintained through different bioprocess. The decrease (*hypouricemia*) or increase (*hyperuricemia*) in the concentration level of uric acid leads to various diseases for instance gout, arthritis, cardiovascular disease, kidney disorder, nephritis, Lesche Nyhan syndrome, and hypertension. Presently, uricase enzymatic assay and the electrochemical practices are very common for monitoring the uric acid levels, but they generally suffers from some serious issues like costlier, low recognition power and so on. Hence, there is a great necessity to produce simple yet straightforward, highly sensitive, nonenzymatic, noninvasive, and reliable techniques for the routine recognition of the uric acid in biological fluids (e.g., *blood and/or urine*). The fluorescence signaling, in particular fluorescence enhancement, is considered as an encouraging surrogate tactic because of its high signal output, besides quick response time.^[^ ^83^, ^84^, ^85^ ^]^ To this context, Kim and his teammates in 2015, have successfully designed and evaluated the features of boron‐dipyrromethene (BODIPY)‐based fluorogenic probe **36** for ‘TURN‐ON’ recognition of uric acid in the serum over a varied concentrations with high selectivity and specificity (**Figure** **9**).^[^ ^86^ ^]^ Uric acid, with its multiple hydrogen bonding sites and functionalized heterocyclic structure, is a suitable guest for HR. Its strong, directed hydrogen bonds and inflexible bicyclic structure enhance binding stability and selective contacts. In contrast, barbiturates, while sharing some structural properties with uric acid, lack the rigidity and organized hydrogen bonding, making them less selective binding partners. The importance of geometric and electrical complementary is highlighted in determining guest selectivity, with uric acid showing more favorable interactions with HR compared to related molecules. Geometry of the receptor **36** and the host–guest complex **35**@**36** were optimized through density functional theory (DFT) involving B3LYP/6‐31G(d). Furthermore, they noticed from the ‘time dependent DFT’ that the transition from the ground state to first singlet excited state for the receptor **36** includes: HOMO‐2 to LUMO, HOMO‐1 to LUMO, and HOMO to LUMO frontier molecular orbitals. From these studies they concluded that HOMO and LUMO exist in the BODIPY moiety, whereas, the electron densities of HOMO‐2 and HOMO‐1 resides in the chelating units, suggesting that photoinduced charge (*electron*) transfer occurs from chelating‐to‐BODIPYT unit. In contrast, after adding uric acid **35** to the host molecule **36**, the molecular orbitals involved in the transitions are: HOMO, LUMO, and LUMO+1, and found that the HOMO was not located at the probe **36**, hence no photoinduced electron transfer (PET) is possible. Interestingly, after uric acid interaction with the probe **36**, the oscillator strength was found to be increased from 0.05 to 0.202, demonstrating increase in the absorbance. The photophysical properties of this event were examined by means of UV–Vis and fluorescence spectroscopy in aqueous solution. The receptor **36** displayed a weak absorption band at 507 nm (*ε* = 13 666.7 M^−1^ cm^−1^), whereas, the addition of uric acid guest (20 μM), increases the absorption intensity which was found to be (*ε* = 23 033.3 M^−1^ cm^−1^) indicating binding of the host **36** with guest **35**. Moreover, a weak emission band (*φ* = 0.004) at 510 nm in the fluorescence spectra was noticed when excited at 475 nm because of the PET from amino‐pyridine entities to the BODIPY unit, which was found to be consistent. Interestingly, after gradual adding uric acid (0–700 μM) to the probe **36**, fluorescence of the system was considerably increased (*φ* = 0.029) displaying worthy linear relationship. Enhancement in the fluorescence was due to inhibition of PET to BODIPY subunit from the amino–pyridine subunits. Furthermore, the 1:1 binding was observed by e Job's plot using changes in the fluorescence of the probe **36** with uric acid guest **35**. The probe selectivity toward the uric acid was noticed by the alternation in emission spectra at 512 nm involving a range of bioanalytes in aqueous solution, like creatinine, xanthene, hypoxanthine, glutathione, cysteine, ascorbic acid, *L*‐tyrosine, homocysteine, glycine, *L*‐serine, *L*‐glutamic acid, *L*‐arginine, and *L*‐aspartic acid. Due to straightforward and nonenzymatic nature of their protocol, and the host–guest stability over the biological pH‐range (5–9), it was proposed that these types of probes could be useful for routine pharmaceutical and clinical analysis of the uric acid in upcoming time.

### 3.3 Anions

**Figure 10:**
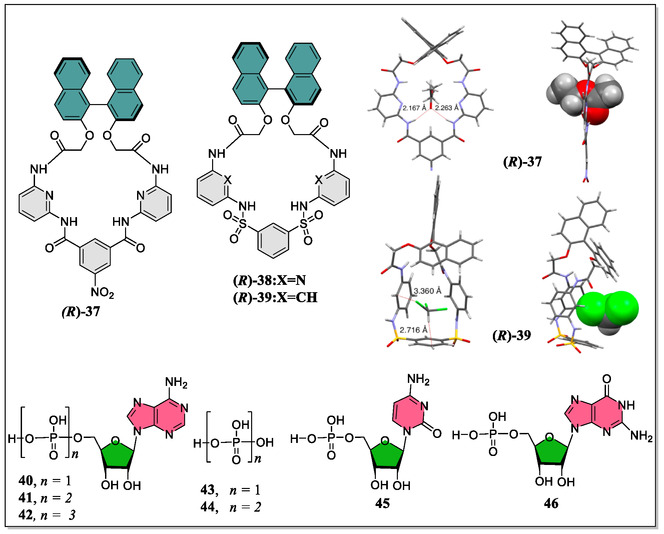
Chemical structures of anionic receptors (**37–39**), and the X‐ray crystal structures of (*R*)‐**37** and (*R*)‐**39** (Reproduced with permission,^[^ ^102^ ^]^ Copyright 2014, American Chemical Society). Structures of the guest molecules whose tetrabutylammonium/sodium salts were used are also depicted.

Since anionic species are omnipresent in nature, playing a myriad of crucial roles in the diverse disciplines of chemistry for instance analytical chemistry, biochemistry, physiology, and pharmaceutical chemistry, and so on.^[^ ^87^, ^88^, ^89^, ^90^ ^]^ Therefore, designing strategies for their recognition or sensing is highly desirable. Unsurprisingly, scientists worldwide are hitting their determinations in this direction. Although, a plethora of anionic receptors involving noncovalent supramolecular interactions, such as electrostatic interaction, H‐bonding, anion‐π interaction, metal coordination, and so on, have successfully been published in the scientific literature.^[^ ^91^, ^92^, ^93^, ^94^, ^95^, ^96^, ^97^, ^98^, ^99^, ^100^, ^101^ ^]^ But this alarming field of research is yet to be sightseen to the advanced level. Toward this point, the research group of Anzenbacher reported three chiral macrocyclic anion receptors **37–39** featuring sulphonamide/amide functional groups in their structures (**Figure** **10**).^[^ ^102^ ^]^ In their study, the authors have recognized and discriminated seven different biologically important phosphate anions in addition to other simple anions, by virtue of these versatile receptors. Interestingly, from the DFT calculations and X‐ray crystallographic studies, they find that these receptors adopt different conformations leading to distinctive binding behavior. First, the anion binding were established by means of electrospray ionization mass spectrometry measurements followed by the NMR titrations, displaying deprotonation of sulfonamide and nitrobenzamide NH‐signals of the receptors (**37–39**) through the addition of simple anions. Simultaneously, they also pointed out different color of solutions upon addition of diverse anions with the receptor **37** as compared to the receptors **38** and **39**, where slight or no color change was experienced. Moreover, upon irradiation of UV light (365 nm), the authors have noticed that the receptor **38** and **39** exhibit blue fluorescence, whereas, the receptor **37** does not display any fluorescence. From the fluorescence experiments, they observed the order of the quantum yields as **39 **> **38 **> **37**. Later on, they turned their attention to study the biological anions, and evaluated the binding affinities of these receptors. From the NMR titrations, they found that **37** does not show any appreciable affinity (*no binding constant was determined*) for cytidine monophosphate (CMP) and adenosine monophosphate (AMP) anions, whereas, **38** and **39** displayed considerable affinities for the same anions. Additionally, they established a sensor array using **38** and **39** capable of differentiating seven phosphates with 100% classification accuracy in an aq. DMSO solution.

### 3.4 Drug Molecules

**Figure 11:**
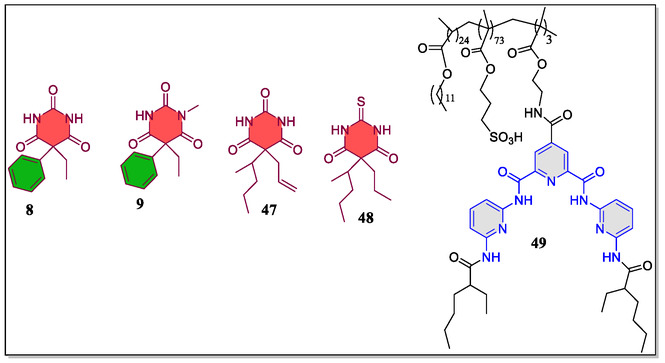
The structures of some guest drug molecules and the Hamilton receptor **49**.

Since artificial receptors display potential applications in biomedicine facilities, selective extraction, membrane transport, and separations techniques.^[^ ^103^, ^104^, ^105^, ^106^, ^107^, ^108^, ^109^, ^110^ ^]^ To this context, the research group of Loizidou et al. have published the molecular docking simulations as well as binding studies of amphiphilic terpolymers, for the selective recognition of structurally discrete drug molecules, such as mephobarbital, phenobarbital, thiopental, and secobarbital in aqueous solutions (**Figure** **11**).^[^ ^111^ ^]^ In these versatile terpolymers, poly(3‐sulfopropyl methacrylate) acts as a hydrophilic component, and the poly(*n*‐dodecyl acrylate) moiety as the hydrophobic component, whereas poly(barbiturate receptor) portion acted as the drug recognition component. The H‐bonding interactions and drug encapsulation studies of the receptor–terpolymer with barbiturates were confirmed by micellar electrokinetic chromatography and by means of the computational docking simulations, resulting comparable binding affinities trends. Finally, they proposed that such types of receptor–polymeric systems might participate in molecular recognition of biologically interesting molecules, and could add value to the selective recognition phenomenon.

### 3.5 Surface‐Confined Systems

**Figure 12:**
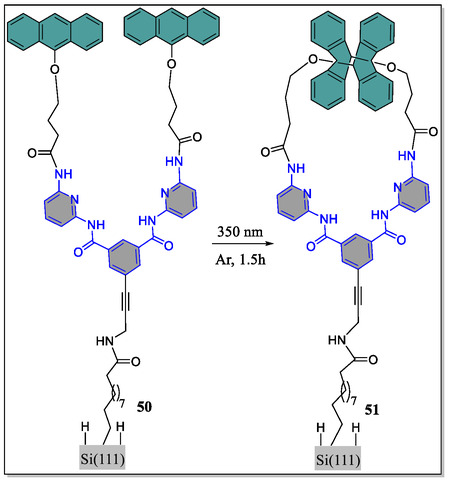
Structural representation of immobilized anthracene‐grafted photoswitchable system.

The molecular recognition on surfaces is an emerging and vastly challenging prospect in the domain of supramolecular chemistry. Remarkably, calixarenes^[^ ^112^, ^113^, ^114^ ^]^ and cyclodextrins^[^ ^115^, ^116^, ^117^, ^118^ ^]^ are the most studied host molecules in this versatile field. But the strength of host–guest supramolecular connections is fundamentally demanding to control. Besides, there are limited reports on the surface‐limited systems in which molecular recognition can be ON‐OFF reversibly. To this frame, Molard et al. have developed a receptor (*host*) for the recognition of barbituric acid (*guest*) whose binding abilities in solution phase could be controlled by means of photoinduced dimerization of the appended anthracene moieties.^[^ ^119^, ^120^ ^]^ On the contrary, immobilization of receptors in self‐assembled monolayers is an area of huge interest among the scientific community. Toward this goal, surface‐immobilized light‐driven reversible molecular receptors (print board), which can be used as “write” and “erase” molecular recognition platform, was reported by the group of Fabre, where they used anthracene‐based receptor monolayers covalently bound to the silicon semiconducting surface (**Figure** **12**).^[^ ^121^ ^]^ Interestingly, this grafted‐anthracene‐receptor ‘open‐form’ was cleanly converted into the ‘closed‐form’ by means of irradiating the immobilized receptor at 350 nm light, confirmed through the infrared IR spectral data, and grafting of both open as well as closed forms did not alter the surface topography to great extent. Moreover, the authors observed from the atomic force microscopy (AFM) spectroscopy, the generation of intense homogeneous molecular films without aggregated islands or significant contamination. Furthermore, an effective charge transport process between the receptors was noticed by the scanning electrochemical microscopy technique, but the kinetics of mediator regeneration was found to be faster in case of the closed‐form. It was further demonstrated by the authors that the light‐activated‐switching receptor display inimitable prospect to outline surface employing the UV‐photolithography by virtue of ‘an optical mask’. Finally, they suggested that in comparison to the earlier studies on gold‐surfaces utilizing photoactive receptors, involvement of silicon‐based surface permits more facetious integration into the electronic molecule‐based devices, and the dissimilar redox responses might be evaluated on an outsized surface area, which may be useful for future analytical applications. Moreover, such types of immobilized receptors could be a versatile platform for the hierarchical assembly of the electro‐ and/or photoactive barbiturate‐attached substrates.

**Figure 13:**
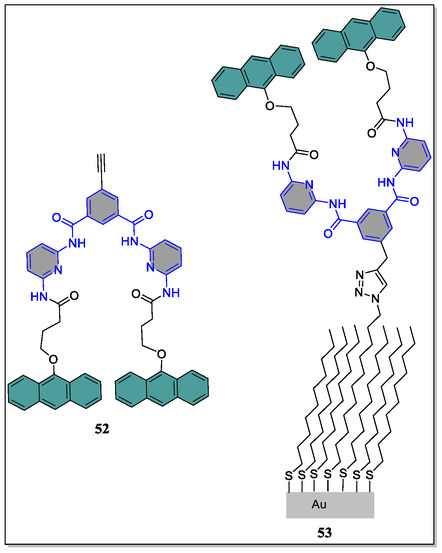
Structural recognition of the photoactive Hamilton‐type receptors **52** and **53**.

In contrast, Bassani and coworkers revealed the construction of anthracene‐based photoactive receptor **52** in addition to its covalent grafting onto the azide‐terminated alkanethiol/gold self‐assembled monolayers by means of popular click reaction (**Figure** **13**).^[^ ^122^ ^]^ Herewith, the immobilized receptor acts as the print board, where H‐bonding recognition can be written with light, thereby generation of the “Read–Write–Erase” molecular print board. Various crucial techniques, such as ellipsometry, surface wetting, AFM, polarization modulation IR reflection‐absorption spectroscopy, were used to characterize the conversion of surface‐attached receptor and its influence on the binding properties with the guest molecule. Photodimerization of the anthracene units by irradiation with light produced the ‘closed‐form’, blocking the gate of the binding site of the receptor. From their studies, they observed that the progression is thermally reversible and the photochemical‐closing was found to be 70%, whereas the thermal‐opening conversion was 100%.

### 3.6 Amino Acids (AAs)

**Figure 14:**
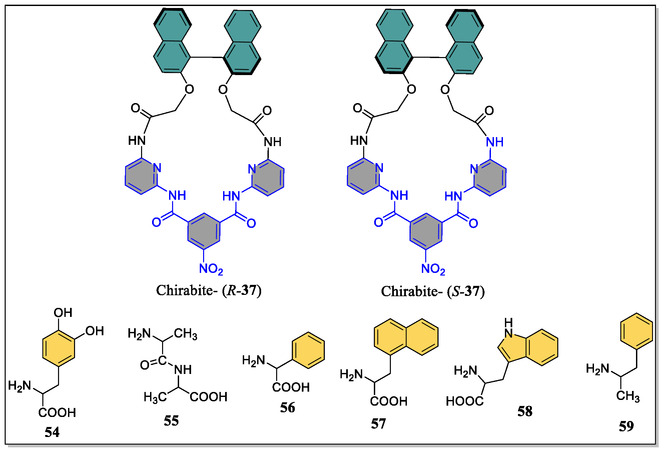
Chemical structures of chirabite‐**37** *(R,S)‐*enantiomers and guest AAs (**54–59**).

**Figure 15:**
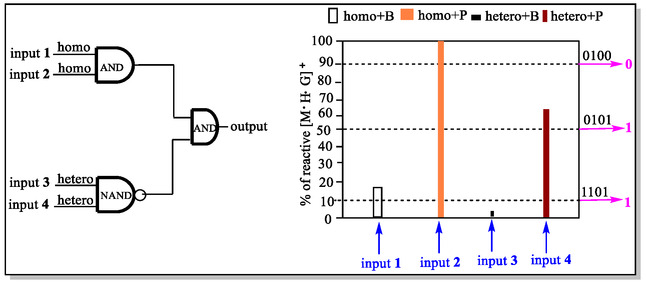
Supramolecular ultra selective multi‐input and multioutput the chemo‐logic gates.

Due to exponential advancements in the drug discovery and developments in recent years, there is an extended demand among the researchers to develop straightforward/economical yet efficient analytical methods for the enantio‐discrimination of valued chiral drugs. Although, a myriad of varied powerful methodologies have already been published in the scientific literature but gas‐phase chiral recognition is nowadays growing with a pace which generally involve the mass spectrometric technique. In this edge, Speranza and teammates have reported kinetic studies of chiral multifunctional macrocycle chirabite‐**37** (**Figure** **14**), which behaves as an efficient and selective platform for chiral AAs in gas phase.^[^ ^123^ ^]^ The AAs in macrocycle host cavity were captured in zwitter‐ionic form via the H‐bonding interaction between pyridine nitrogen atoms and that of AAs proton, but the same types of interactions were not detected for dipeptides/mono‐functional amines. Interestingly, these dipeptides/mono‐functional amines were easily kicked‐off from the corresponding complexes with AAs not depending upon the basicity of the amines and/or the configuration of AAs, thereby generation of the multi‐input and multioutput highly selective chemo‐logic gates (**Figure** **15**).

### 3.7 Racemic Guest Molecules

**Figure 16:**
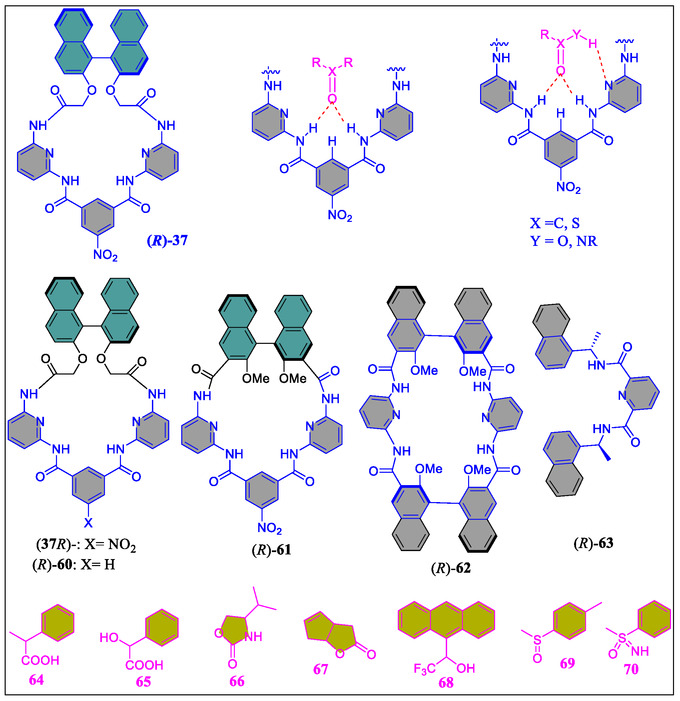
Structure of some guests (**64–70**) and the receptors *R*‐**37**, *R*‐**60**, *R*‐**61**, *R*‐**62**, and *R*‐**63**.

On the contrary, in the year of 2006, Ema et al. have reported quite simple yet practical route for assembling a C2‐symmetric chiral bifunctional macrocyclic system (*R*)‐**51** having several H‐bonding acceptor/donor sites (**Figure** **16**).^[^ ^124^ ^]^ Interestingly, this versatile receptor acts as a novel chiral shift reagent for diverse chiral molecules, like alcohol, carboxylic acid, lactone, oxazolidinone, sulfoxide, isocyanate sulfoximine, and epoxide functionality, as shown in Figure 16. The chiral discrimination capability of this novel host was measured through NMR titrations in CDCl3 solvent displaying (1:1) host:guest binding, which was confirmed by the Job's plots, also known as the method of continuous variation. The binding constants calculated by nonlinear least‐squares method with diverse guests are tabulated in the **Table** **3**. More interestingly, as can be inspected from the same table, good enantiomeric discrimination was realized in several cases. The larger binding constant value suggests that the groups are focused inside the host cavity, and the order for few guests was observed as: sulfoxide ∼ carboxylic acid > oxazolidinone ∼ sulfoximine > lactone, reflecting the number of hydrogen bonds between the host and guest molecules with the exception of sulfoxide and carboxylic acid. From the observation, it was noted that, in case of two‐point interaction systems, the sulfoxide **69** is strongly bounded as compared to the lactone **67**. Whereas in three‐point interactive systems, the carboxylic acid **64**, affianced tightly than oxazolidinone **66** and sulfoximine **70**. Further, they noticed that binding of (*S*)‐**69** and (*S*)‐**70** are 4.3‐ and 4.9‐fold higher, respectively, in comparison to the (*R*)‐**69** and (*R*)‐**70** (Table 3). Importantly, the authors found NMR signals in the complex formed between host and guest, which prove that the receptor reagent is appropriate for high‐field NMR spectrometer. Remarkably, the urgency of determining enantiomeric purity while asymmetric synthesis, diverse chiral shift reagents, or solvating agents for instance lanthanide complexes, crown ethers, cyclodextrin, porphyrins, calixarenes and so forth, have already been clustered into the literature. But due to some shortcomings with these reagents like signal expansion at higher magnetic field, paramagnetic metal, or precipitation through ligand exchange in the presence of lanthanide complexes. In most of the cases, the stoichiometric amount of reagent is required for signal splitting, besides crown ethers are operative only for amines. Therefore, keeping in mind some vital points related to the effectiveness of these reagents, such as high sensitivity, high splitting ability, signal sharpness, wide detection window, versatility, and synthetic accessibility, one year later to the earlier report, the same group has exposed, a series of bifunctional macrocycles **37**, **60–62** and diamide‐based receptor **63** (Figure 16).^[^ ^125^ ^]^ From their studies, they observed that bifunctional macrocycle **37** acts as the best chiral solvating agent with various chiral substances, like oxazolidinone, carboxylic acid, sulfoximine carbonate, alcohol, lactone, sulfinamide, sulfoxide, isocyanate, or epoxide functionalities (Figure 16). Moreover, macrocycle **37** displayed high sensitivity, as in the case of sulfoxide guest only 5 mol% (i.e., 69 μg) was sufficient for nice splitting of the enantiomeric sharp signals, thereby seemly for high‐field NMR spectrometers. Interestingly, among all the systems reported by the authors, receptor **37** showed worthy features, like high splitting ability, broad detection window, great versatility, high sensitivity, nice signal sharpness, and stunning synthetic accessibility. The degree of enantioselectivity and binding capacity of these systems was justified by them based on the suitably oriented cavity as well as H‐bonding sites. Furthermore, the comparative study of **37** and **61** reveals the reputation in orientation of the binaphthyl units and the orthogonal outlook of the binaphthyl subunit in **37**, effectually fetches the differential ring‐current upshot on the chiral guest molecules, which leads to high degree of chiral discrimination in the NMR results.

**Table 3:** Binding constants and recognition energies of (*R*)‐**51** for various chiral guests.

Guest	*K*_a_ [M^−1^]a)	ΔΔ*G°* [kcal mol^−1^]b)
(*R*)‐**64**	1670	−0.35
(*S*)‐**63**	3050	
(*S*)‐**66**	510	+0.35
(*R*)‐**66**	280	
(1*R*, 5*S*)‐**67**	51	+0.26
(1*S*, 5*R*)‐**67**	33	
(*R*)‐**69**	610	−0.85
(*S*)‐**69**	2600	
(*R*)‐**70**	170	−0.93
(*S*)‐**70**	830	

a) In CDCl_3_ at 22 °C.

b) Chiral recognition energy calculated from −RTln{K_a_(S)/K_a_(R)}.

On the contrary, V‐shaped prearrangement of the 2,6‐diacylaminopyridine units in case of **37** was noticed to be much more favorable for binding diverse guests as compare to the parallel arrangement of the two binding units in **62**. The better capability of **37** than **60** was due to the presence of NO_2_ group which not only enhance the binding capability but also increases the degree of enantioselectivity. More importantly, as the compound **37** is nowadays commercially available, unsurprisingly it would contribute largely to the high‐throughput scientific research in the domain of asymmetric synthesis in future studies.

**Figure 17:**
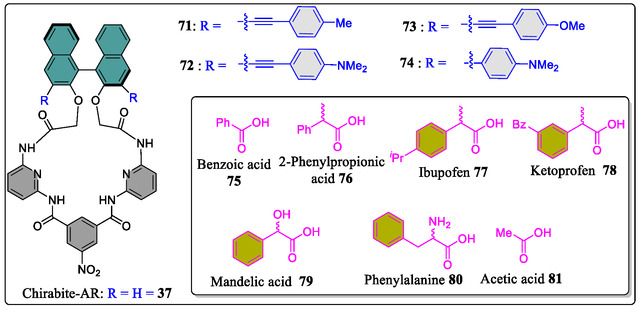
Structures of the Hamilton host (**71**, **72**, **73** and **74**) and guests **65**, **75–81** molecules.

The groups of Ema and Anzenbacher designed and constructed four macrocyclic chemosensors (**71–74**) having BINOL auxiliary attached with the Hamilton‐based architecture.^[^ ^126^ ^]^ They are the congeners of chirabite‐AR (**37**) in which binaphthalene units at the 3,3‐positions were substituted with conjugated aryl‐derivatives to enhance the chiral induction in one hand and fluorescence modulation on the other hand (**Figure** **17**). Importantly, these versatile macrocyclic fluorescent chemosensors displayed good ability to discriminate the carboxylates such as enantiomers of mandelate, ibuprofen, phenylalanine, ketoprofen, and 2‐phenylpropanoate in a stereoselective as well as high‐throughput manners. The chiral carboxylates binding studies were confirmed by means of ESI‐mass spectrometry, DFT calculations, NMR titrations, and X‐ray crystallography. Importantly, the quantitative investigation of enantiomeric composition of ketoprofen, ibuprofen, and phenylalanine demonstrates that the sensors precisely detect mixtures with erratic enantiomeric excess (ee) and decorously envisage the ee of unknown compounds. Moreover, the authors have also observed that the enantiomers of these analytes were determined with 100% accuracy. Furthermore, these authors suggested that as chirabite‐AR can discriminate the enantiomers of the vital compounds like lactones, carboxylic acids, alcohols, oxazolidinones, sulfoxides, sulfoximines, epoxides, and isocyanates. The newly developed fluorescent derivatives or tailored congeners could be having a wide applicability in microarray sensing. In another experiment, Ema's research group has reported superbly modified Chirabite‐AR receptor for the tuning and evaluation of chiral recognition through multiple H‐bonding sites.^[^ ^127^, ^128^ ^]^

**Figure 18:**
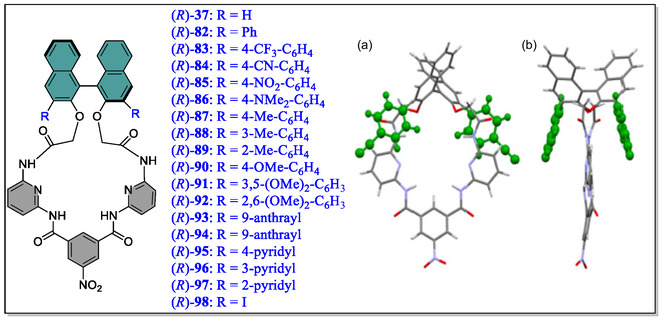
Chemical structures of (*R*)‐**37**, (*R*)‐**82**, (*R*)**83**–(*R*)**98**, and X‐ray crystal structure of (*R*)‐**84** a) Front view and b) side view. Reproduced with permission,^[^ ^129^ ^]^ Copyright 2018, American Chemical Society.

**Figure 19:**
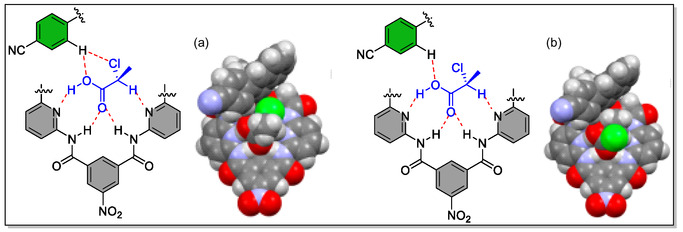
Optimized structures for the complexes of (*R*)‐**84** with a) (*S*)‐CPA and b) (R)‐CPA. DFT calculations were performed with Gaussian 16 at the B3LYP/6‐31G(d) level. Reproduced with permission,^[^ ^129^ ^]^ Copyright 2018, American Chemical Society.

As 3,3′‐positions in binaphthyl system are considered as hot spots for modifying the shape as well as size of the host macrocyclic cavity. Toward this strategy, two synthetic routes, either early stage or late stage have been chosen by the synthetic chemists to install substituents at these particular positions. To this concern, the Ema's group has synthesized various chiral macrocyclic receptors (**82–98**) consisting of multiple H‐bonding sites via Suzuki–Miyaura cross‐coupling reaction (**Figure** **18**).^[^ ^129^ ^]^ Among them, **84** consisting of 4‐cyanophenyl unit was found to be best chiral solvating agent CSA for the 2‐chloropropionic acid (CPA) in CDCl_3_ solvent and both the quarter (H) as well as doublet (CH_3_) signals of the benchmark analyte CPA were completely splited. Furthermore, from the NMR titration, the authors observed the most enantioselective binding constants (*K*_a_) in the case of *R*‐**84,** where they found *K*_a_(*S*)/*K*_a_(*R*) = 5.4). Moreover, they observed that the value of binding constants (*K*_a_) of (*R*)‐**84** for the guest (*S*)‐CPA (5900 M^−1^) is far larger than the (*R*)‐**37** for the (*S*)‐CPA (3080 M^−1^), thereby suggesting attractive interaction of (*S*)‐CPA with 4‐cyanophenyl group of (*R*)‐**84**. This vital attraction was confirmed by the DFT calculations as (C—H···Cl—C—H‐bond), that is, interaction between one of the *meta*‐hydrogen of the 4‐cyanophenyl group and Cl atom of (*S*)‐CPA (Figure 18). Moreover, from the X‐ray crystal structure, it was analyzed that the 4‐cyanophenyl group come into the close contact with the upper amide group having distance (3.24–3.42 Å) between the centroid of the benzene ring and the amide nitrogen atom. In short, the two hydrogen atoms *meta*‐ and cyano group are directed toward the cavity in the receptor **84** (**Figure** **19**). Hence, this valued investigation showcases the supremacy and the usefulness of a specific macrocyclic receptor (CSA) from a library of compounds for a particular analyte.

## 4 Solubilization and Separation of the Covalently Sidewall‐Functionalized Single‐Walled Carbon Nanotubes (SWCNTs)

**Figure 20:**
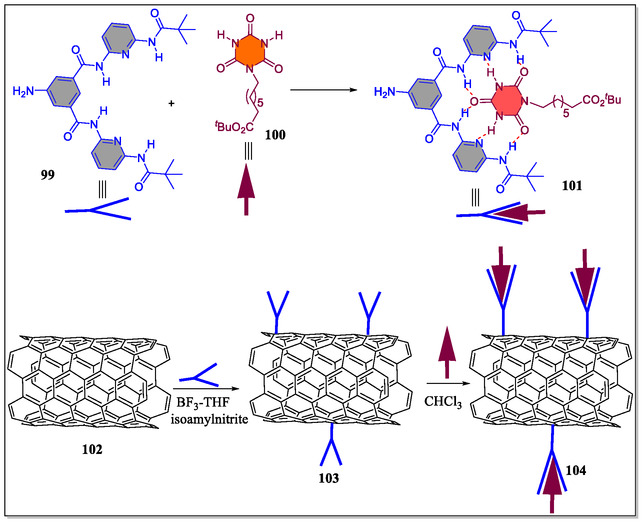
Functionalization of SWCNTs with cyanuric acid derivative **100** and Hamilton receptor **99**.

Functionalized SWCNTs need to be soluble, which are considered as effective for developing sensors, electronics, and nanocomposites. This involves the addition of functional groups through chemical reactions to improve dispersibility in solvents. Common methods include amidation reactions or diazonium chemistry to attach carboxyl, amine, or aryl groups. These groups enhance solubility and compatibility with different matrices, enabling the production of advanced materials and devices on a large scale. Separation techniques, such as selective precipitation, size‐exclusion chromatography, and density gradient centrifugation, are crucial for ensuring consistent properties of covalently functionalized SWCNTs. By sorting SWCNTs based on length, diameter, or electrical characteristics, these methods enhance performance reliability. The efficiency of separation processes is influenced by the functional groups introduced during covalent modification. Optimal solubilization and separation methods are essential for maximizing the potential of functionalized SWCNTs in nanotechnology applications.^[^ ^130^, ^131^ ^]^ Scientists worldwide are highly interested in the exfoliation of bundled SWCNTs besides stabilization of the individualized moieties because of their diverse applications in high‐performance materials. This is a central requirement for a competent functionalization in addition to sorting of the SWCNTs with regard to their electronic type, diameter, and helicity.^[^ ^132^, ^133^ ^]^ To this context, in recent years, researchers across the globe have successfully functionalized SWCNTs by means of different strategies, but involving diazonium compounds is found to be a very useful functionalization tactic that allows the installment of diverse functional groups onto the SWCNT framework.^[^ ^134^, ^135^, ^136^ ^]^ On the other front, generally, covalent attachment of the solubilizing addends to the nanotube scaffold offers an opposite scenario for the easy and fast separation of selectively functionalized nanotubes on the basis of simple yet effective extraction steps. Interestingly, for increasing the SWCNT dispersibility to a greater extent, supramolecular approaches have also been fruitfully investigated.^[^ ^137^, ^138^, ^139^ ^]^ Toward this perspective, Hirsch and teammates have reported a novel efficient way based on supramolecular approach for the facile solubilization and separation of the covalently functionalized SWCNT utilizing diazonium functionalization sequence (**Figure** **20**).^[^ ^133^ ^]^ Importantly, the driving force for deeply improved dispersibility for the extended‐time stability of the individualized SWCNTs. The supramolecular complex formation between the Hamilton receptor with the cyanuric acid derivative in chloroform was confirmed by the UV/Vis‐NIR and AFM analysis. The covalent functional derivatives of metallic carbon nanotubes were investigated by means of Raman microscopy, and fluorescence and UV/Vis‐NIR spectroscopy besides mass spectrometric coupled thermogravimetric analysis. The author's prospect that intense studies of this solution‐based separation technique will permit an improvement of functionalized metallic SWCNTs in preferred solvent(s) with appropriate cyanuric acid derivatives. They further suggested that the logical supramolecular functional SWCNTs, cyanuric acid derivatives, and Hamilton receptor could be used to engender fairly intricate architectures having promising applications in electronic materials in future studies.

## 5 Hamilton as Multitype Receptor System

**Figure 21:**
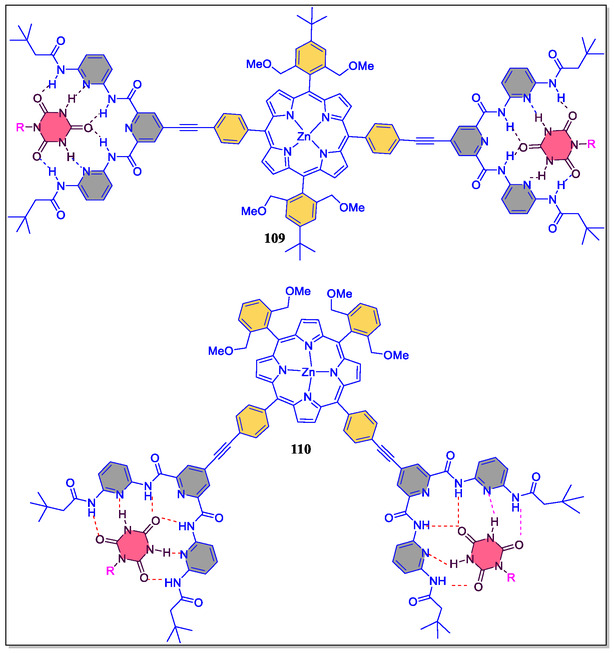
Supramolecular complexes of Zn‐porphyrins and Hamilton receptor **109** and **110**.

**Figure 22:**
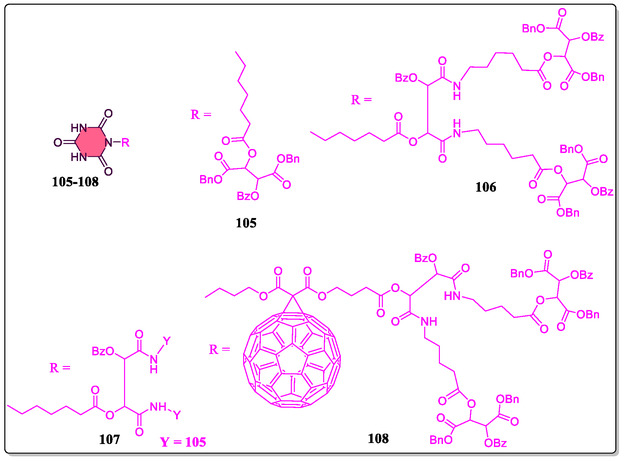
Chemical structural view of diverse guest molecules **105–108**.

In a separate report, Hirsch's group has synthesized supramolecular building blocks *cis‐* **109**/*trans‐* **110** zinc‐porphyrin‐based Hamilton receptors, and also investigated their photophysical and aggregation properties (**Figure** **21**).^[^ ^140^ ^]^ The association constants for the host–guest complexation were investigated by NMR studies and fluorescence spectroscopic titrations. From the NMR titration results, it was observed that the main stability of the complex and supreme cooperativity for 1:2 aggregates concerned the first generation depsipeptide dendron **105**. The self‐assembly comparative study of the first‐to‐third generation complexes of *cis‐* and *trans‐*geometry confirms that *trans‐isomer* is highly pronounced as compared to the *cis*‐substituted analog. On the contrary, the steric loading of the ligands increases by increasing the number of depsipeptide dendrons **105**, **106**, and **107**, which leads to decrease of association constants *K*_2_. The graphical clarifications of all possible **109·105**_***n***_ (***n*** **= 1–2**) complexes in solution exposed that the **105·105**_**2**_ complexes conquer over **105·105**_**1**_ aggregates at low concentrations (**Figure** **22**). Whereas, the **105·105**_**2**_ assemblies, high bulky third generation dendron **107** was found to be very less around concentration 45%–70%, whereas the concentration of **105·105**_**2**_ were found between 95% and 100%. The Job's plot obtained from ^1^H‐NMR titration experiments confirmed a 1:2 stoichiometry of the first‐to‐third generation aggregates. Importantly, the association constants from the fluorescence titrations were in great agreement to that obtained with ^1^H‐NMR titration analysis. Furthermore, from the photophysical studies, for instance, time‐resolved and steady‐state fluorescence on the femtosecond/nanosecond time‐scales, the authors obtained validation of the nature of electron D–A interactions.

## 6 Mechanically Interlocked Molecules (MIMs) Based on Hamilton Receptor

**Figure 23:**
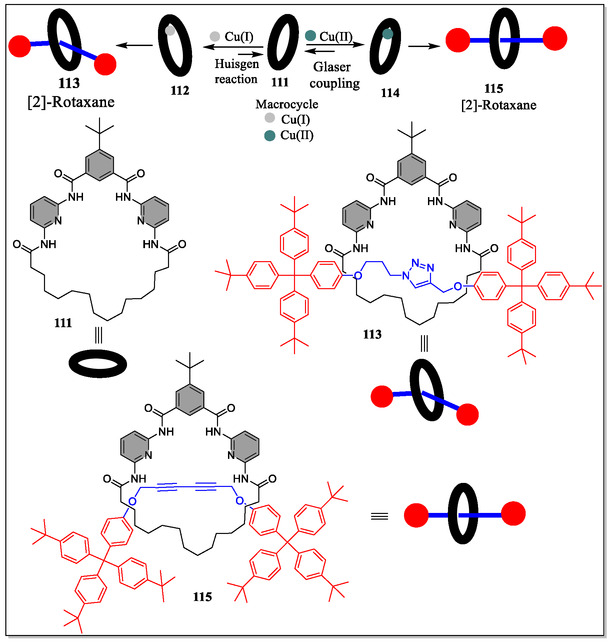
Pictorial representation for the template synthesis of [2]‐rotaxanes **113** and **115** via the Huisgen cycloaddition and Glaser‐coupling reactions, respectively.

MIMs are potentially significant class of supramolecular structures and their inimitable electrochemical as well as physiochemical properties makes them potential candidates for diverse valuable applications from materials science, polymer chemistry, medicine, nanotechnology, to the catalysis, optical, drug delivery, electrochemical sensors and so on. On the contrary, supramolecular rotaxanes display significant applications in the construction of polymeric materials (e.g., molecular muscles, hydrogels, micelles and nanoparticles),^[^ ^141^ ^]^ molecular devices, e.g., logic gates,^[^ ^142^ ^]^ nano‐valves,^[^ ^143^ ^]^ switches,^[^ ^144^ ^]^ and sensors,^[^ ^145^ ^]^ and artificial molecular machines, nanomotors,^[^ ^146^ ^]^ molecular ratchets,^[^ ^147^ ^]^ or elevators^[^ ^148^ ^]^ in addition to strong impact in the domain of biomedical and pharmaceutical sciences.^[^ ^149^ ^]^ Therefore, a number of interesting interlocked building blocks have been explored in the past number of years whose design depends upon the utilization of comparatively small binding/templating units.^[^ ^150^ ^]^ Among which, rotaxanes are class of MIMs with cyclic and linear moieties, coordinated together in threaded pattern through noncovalent interactions. However, rotaxanes lacking bulky stopper at terminal points are called as pseudorotaxanes, whereas rotaxanes with single stopper are recognized as the semirotaxanes. The inclusion of interesting functional groups, gives birth to complex systems ranging from simple [1]‐rotaxanes,^[^ ^151^ ^]^ [2]‐rotaxanes, oligo‐rotaxanes,^[^ ^152^ ^]^ to daisy chains,^[^ ^153^ ^]^ polyrotaxanes.^[^ ^154^ ^]^ Keeping the potential applications of MIMs in mind, Tron et al. in 2016, have reported the complexation of Cu(I) with Hamilton receptor and its subsequent use in an active template [2]‐rotaxanes formation through the Huisgen cycloaddition and Glaser‐coupling reactions (**Figure** **23**). Macrocyclic version of the Hamilton receptor **111** was found to bind the copper (I) with binding constant, *K*_a_ = 60 000 M^−1^ in chloroform. From the results, it was observed that the rotaxane **112** obtained via click reaction can rebind copper (I) in chloroform solvent less strongly than to the parent macrocycle, and does not display any H‐bonding with *N*,*N*‐trimethyleneurea or barbital. Whereas, the rotaxane **115** obtained via the Glaser reaction, bind with barbital and *N*,*N*‐trimethyleneurea with binding constants of about 8 000 and 110 M^−1^, respectively, in CHCl_3_. Hence, shifting of selectivity or poorer guest selectively, with respect to the parent macrocycle upshots from a less distinct receptor site.^[^ ^155^ ^]^

**Figure 24:**
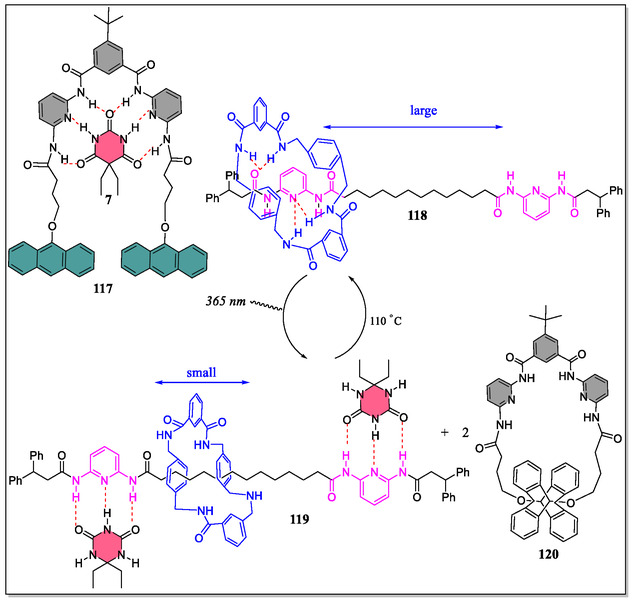
Representation of the supramolecular super‐system and the binding with barbiturate.

A novel photoswitchable multiple H‐bonding molecular [2]‐rotaxane‐based receptor acting as an molecular effector to control the ring gliding between the two‐stations, in which barbiturate messenger was found to be easily and reversibly released/captured, has been exposed by the research groups of Berna and McClenaghan (**Figure** **24**).^[^ ^156^ ^]^ The photoirradiation of **117** effects the [4π + 4π] anthracene cycloaddition reaction giving **120**, and reproduce the receptor site which are not suitable to accommodate **6**, thereby, promoting photorelease and transfer of self‐directed effector **6**. Although, the benzylic amide rotaxane **119** binds with **6** to form 1:1 and 1:2 complexes with *K*_a_ **118** (1:1) = 787 M^−1^ and *K*_as_ **118** (1:2) 108 M^−1^, respectively. In contrast, the photoactivity of molecule **117** molecule were studied by McClenaghan, and confirmed that **117** displayed binding with barbital having *K*_a_ **(117)** = 38 000 M^−1^ through six H‐bonds.^[^ ^156^ ^]^ The di(acylamin)pyridine‐based [2]‐rotaxanes (DAP) displayed dynamic behavior due to the presence of two *endo*‐pyridine rings in tetralactam macrocycle. This dynamic behavior leads to reduction in the spinning rate around the thread due to existence of stronger intercomponent H‐bonding interactions. The authors demonstrated that the coconformational exchange can be activated by molecular recognition utilizing *N*‐hexylthymine. However, the complexation of thymine with DAP binding site support the formation of ‘*S*’‐shaped conformer, in which the macrocycle concurrently display interactions with both DAP‐domain as well as amide station. Interestingly, from the experimental studies, these workers reveal that the guest binding at both rotaxane diamidopyridine sites directly influences the amplitude of the ring shuttling.^[^ ^157^ ^]^ The barbiturate was found to be residing quasi‐exclusively at the receptor **117**, and photoirradiation provoked the release of **7**, thus, chemical transfer, and effective chemical communication with the rotaxanes. In this article, they have monitored the entire process by means of UV−Vis spectroscopy and ^1^H‐NMR technique.

**Figure 25:**
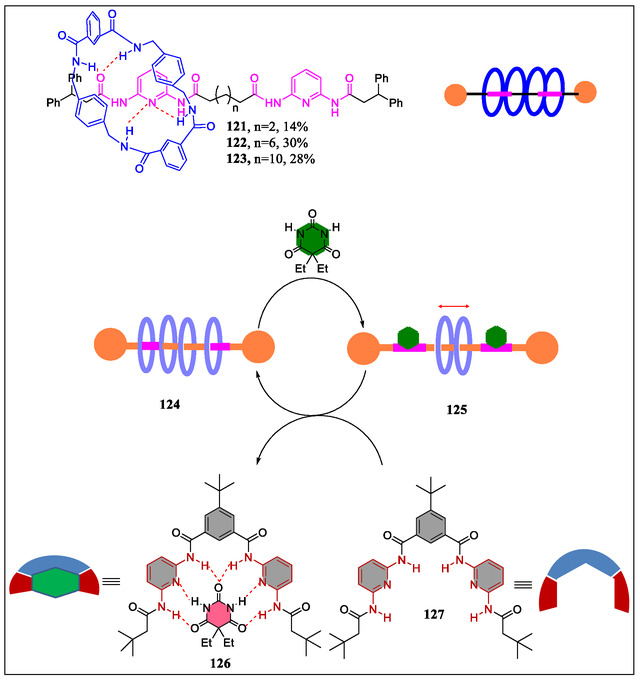
Reversible control of the translational motion in the interlocked **125** through the formation of stronger ADA–DAD bonding pattern.

In 2014 Cuezva et al. have reported a tailor‐type rotaxane, which facilitates induced response to addition of small molecules by five‐component clipping reactions in better yields.^[^ ^158^ ^]^ From the solid state observations, it was observed that the involvement of pyridine nitrogen in the stabilization of mechanical bond was due to intramolecular H‐bonding between thread and benzylic amide macrocycle (**Figure** **25**). However, in case of rotaxanes possessing two active binding sites, the competitive association with external binders having an array of H‐bond acceptor and donor site potentially blocks the binding sites of thread, which in turn restricts the amplitude of the ring motion. Whereas, the original state can be restored by employing a new detection event using addition of preorganized bis(di‐(acylamino)pyridine) resulting in the formation of stronger ADA–DAD complexes with external H‐bonding. In this article, the authors commented that these interlocked molecules will be promising complexes for the design and synthesis of new biomimetic complex devices and supramolecular catalysts for future research.

**Figure 26:**
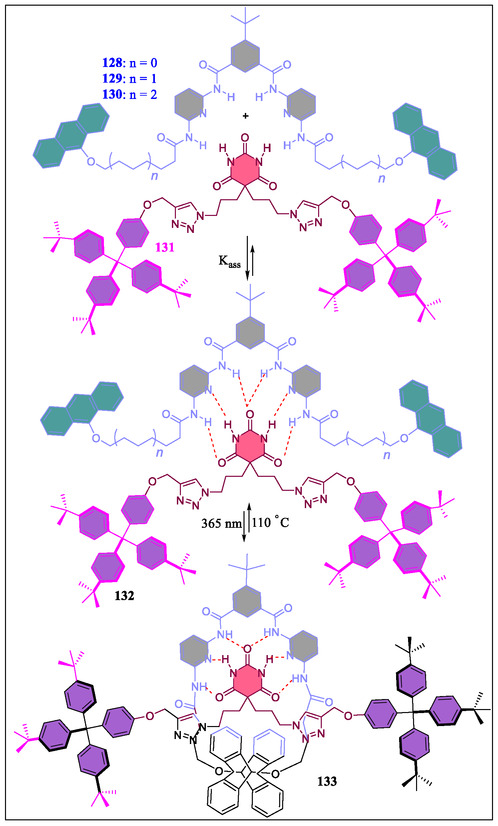
Complexation of Hamilton receptors **132** and **133** with the barbital‐based guest **131**.

**Figure 27:**
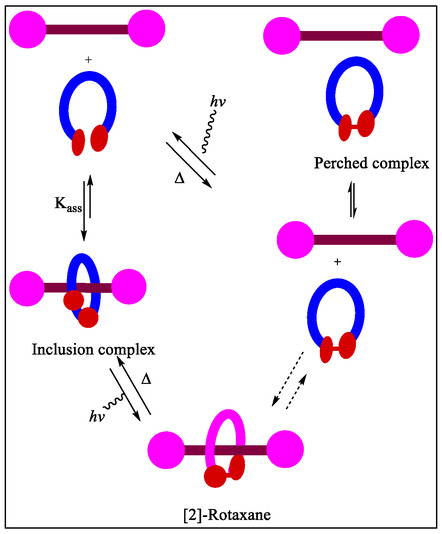
Diagrammatic representation of the [2]‐rotaxane formed by photoclipping of an acyclic receptor with anthracene terminal groups as stoppered thread and disassembly by thermal retro‐cyclomerization.

**Figure 28:**
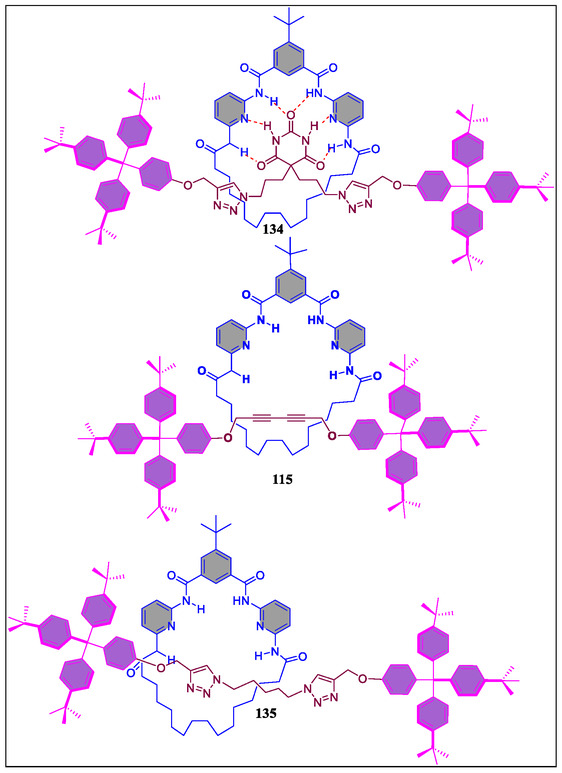
Structural representation of mechanically interlocked super structures **134**, **115**, and **135**.

In 2015, Tron et al. have reported the development of H‐bonding between stoppered molecular thread **131** with barbiturate and photoactive acyclic Hamilton receptor **129–130** (**Figure** **26**). The consequent light‐driven ring‐closure results in the formation of [2]‐rotaxane with thermal reopening to reset systems (**Figure** **27**). The photoirradiation of H‐bonded molecular complex containing the acyclic stoppered thread **128** with barbiturate moiety and doubly anthracene‐terminated acyclic Hamilton‐like receptor **130**. From the NMR spectroscopy and molecular modeling, it was observed that the isolated interlocked photoproduct (*Φ* = 0.06) of the [2]‐rotaxane with dimerized anthracene is in head‐to‐tail fashion. Diverse nature was examined by irradiating homologous molecular complexes **132** and **133**. The kinetically labile rotaxane was produced on irradiating complex **133**. On the contrary, potentially stable [2]‐rotaxane was obtained on irradiating **133** displaying capturing power, thereby reorganizing the complex with fatigue (38%) after four irradiation‐thermal reversion cycles.^[^ ^157^ ^]^ In contrast, the same group has synthesized interlocked structures **115** and **134** (**Figure** **28**), which demonstrates the potential applications for H‐bonding motif in various functional interlocked systems. Recently, McClenaghan and coworkers have reported the study of a macrocycle shuttling in rotaxanes containing HR **115** and **134** (Figure 28).^[^ ^159^ ^]^ The dramatic changes in macrocycle shuttling rates at 25 °C were observed from different stations in the order of barbiturate < triazole < phenol. From the results, the authors revealed that these types of rotaxanes are applicable in introducing predetermined macrocycle shuttling rates in 2‐station rotaxanes.

**Figure 29:**
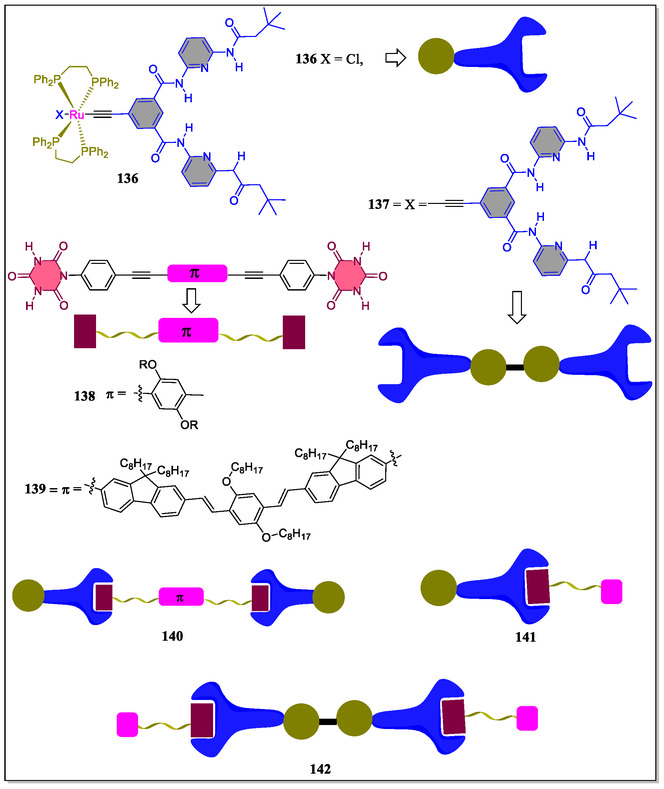
Representation of the H‐bonded mono‐ and bifunctional supramolecular complexes.

**Figure 30:**
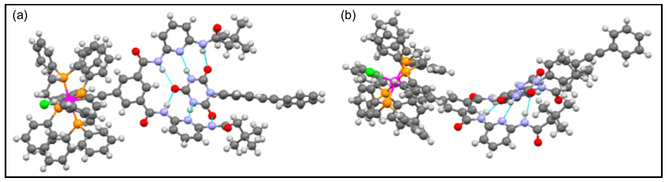
X‐ray crystal structural view of the complex **142** a) top view; b) side view. Reproduced with permission,^[^ ^160^ ^]^ Copyright 2013, American Chemical Society.

Synthesis of a series of hydrogen‐bonded supramolecular complexes based on bulky mono‐ and bifunctional *trans*‐alkynylbis(1,2‐*bis*‐(diphenylphosphino)ethane)ruthenium(II) complexes and π‐conjugated blocks of 2,5‐dialkoxy‐*p*‐phenylene have been successfully reported by Fillaut and coworkers (**Figure** **29**).^[^ ^160^ ^]^ The self‐assembly of these supramolecular complexes were obtained by introducing the Hamilton receptor and complementary cyanuric acid derivatives at the terminal positions. From the results of ^1^H‐NMR titration experiments and Job's plot analysis, it was revealed that the complexes **141** and **142** are present in 1:1 and 1:2 stoichiometries, respectively. The association constant of complexes **140**, **141**, and **142** were found as log *K*_1_ ≈ 4, log *K*_2_ ≈ 4.7, *K*_1_ ≈ 4, and log *K*_1_ *K*_2_ ≈ 8.7, respectively. These association constant values suggests that the presence of two active receptor sites in the receptor system **137** acting independently and forms the self‐assembly and disassemblies. The incorporation of 2,5‐dialkoxy‐*p*‐phenylene cores resulted in sensing of 2,4‐dinitrotoluene in chloroform solvent through the fluorescence quenching experiment. From the X‐ray studies, it was revealed that the complex **142** form stable single crystal in THF, and further perceived that the Hamilton receptor is twisted in nature with respect to aromatic core (**Figure** **30**a–b). From the overall results, it was clear that both the self‐assemblies and other modifications in the complementary blocks are considered as promising materials for responsiveness of electron‐rich species, 2,5‐dialkoxy‐*p*‐phenylene spacers toward nitro aromatics.

**Figure 31:**
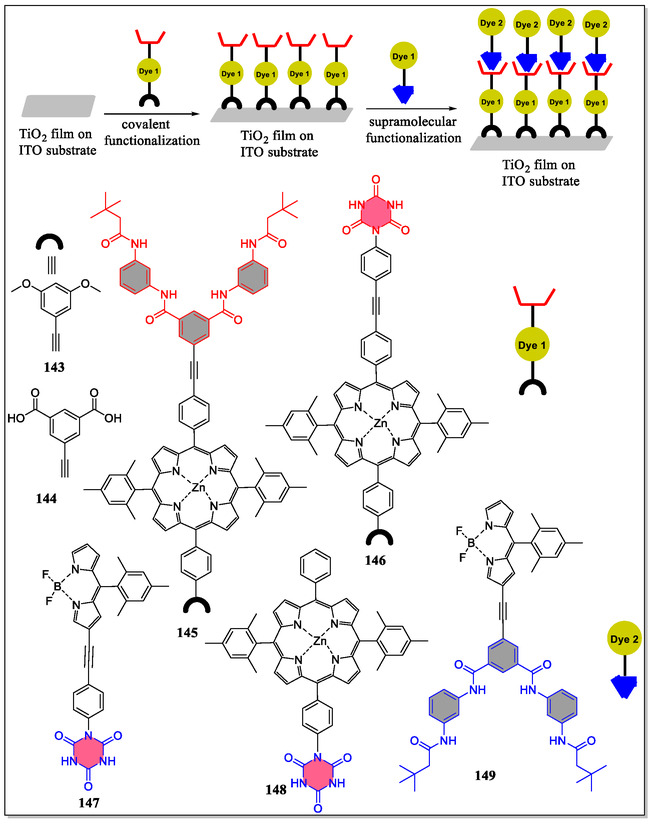
Diagrammatic representation for the functionalization of doctor‐bladed TiO_2_ films with dyes through covalent and noncovalent ways.

Recently, Hirsch and coworkers have reported controlled deposition of BODIPY and porphyrin–porphyrin bilayers onto the surface of doctor‐bladed TiO_2_ films through combination of both covalent and noncovalent self‐assembly (**Figure** **31**).^[^ ^161^ ^]^ Various Hamilton‐receptor‐based porphyrins and/or porphyrin‐based cyanurates are one or two carboxylates as TiO_2_ anchors were designed and prepared through multisteps organic syntheses. In this article, complemented supramolecular recognition motifs were observed by considering porphyrins and BODIPY. Moreover, the coating strategy synergizes the reimbursement of potential covalent anchoring of first dye layer, that is, (metallo)porphyrin, with noncovalently attachment of second dye layer, that is, (metallo)porphyrin or BODIPY. Interestingly, the authors noticed that **TiO**_**2**_ **·145·146** displayed higher DSSC efficiencies (43%). From the results of transient absorption spectroscopy measurements, it was discovered that only **TiO**_**2**_ **·145·147** displayed the spectroscopic features of one‐electron oxidized form of (metallo)porphyrin **145** and one‐electron oxidized BODIPY **147**. Thus, **TiO**_**2**_ **·145·146** showed an intramolecular CT process from **147** to **145** following the initial interfacial charge injection from **145** into TiO_2_.

**Figure 32:**
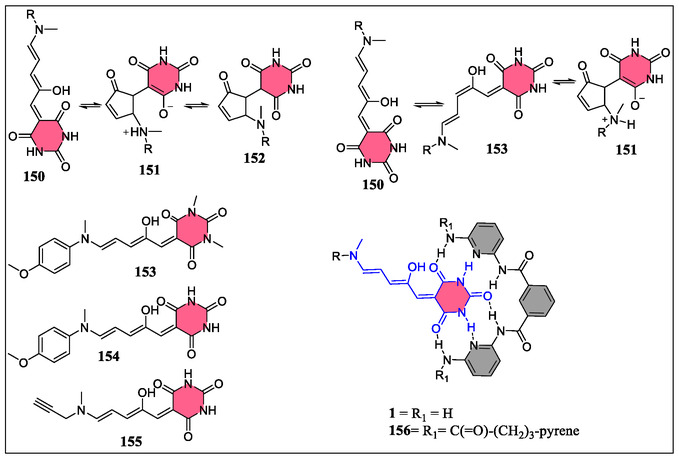
Structural representation of common isomers of DASAs and host–guest contacts between DASAs and HR.

On the contrary, Beves and coworkers have reported the synthesis of D–A Stenhouse adducts (DASAs) with free amido units onto the barbituric acid acceptor group acting as a H‐bonds donor (**Figure** **32**).^[^ ^162^ ^]^ The presence of different active H‐bonding sites in DASAs featuring barbituric acid groups, displayed the switching properties in the presence of Hamilton receptor, which were controlled through the host–guest supramolecular interactions. The switching properties were quantified due to thermal isomerization of DASAs (**153–155)**, which was occurred through a stepwise linear‐enol‐keto mechanism between ring‐closing and tautomerization reactions (Figure 32). Transient absorption spectroscopic study revealed that sufficiently high thermal barrier between intermediate (**153**) and the stable linear isomer (**150**) is critical for allowing the formation of a cyclic isomer, which may be the determining factor for switching properties. However, the binding of DASA **154** with **1** or **156** receptors stabilizes the linear isomer (**150**) as compared to the cyclic isomer (**151**). The decrease in barrier between the intermediate (**153**) and linear isomer (**150**) as compared to intermediate (**153**) and cyclic form (**151**) was also observed. In contrast, Altintas et al. have manufactured a *α,ω*‐hydrogen acceptor/donor single chain self‐assembly of a polymeric system obtained from the cyanuric acid and Hamilton wedge.^[^ ^163^ ^]^ From the analysis of ^1^H‐NMR and DLS, entropically stronger H‐bonding contacts between the *α*‐donor and *ω*‐acceptor were observed, which leads to a circular single chain self‐assembly. The formation of this circular single chain assembly in the polymer system mostly favors at concentrations of ≈0.5 mM in CDCl_3_.

**Figure 33:**
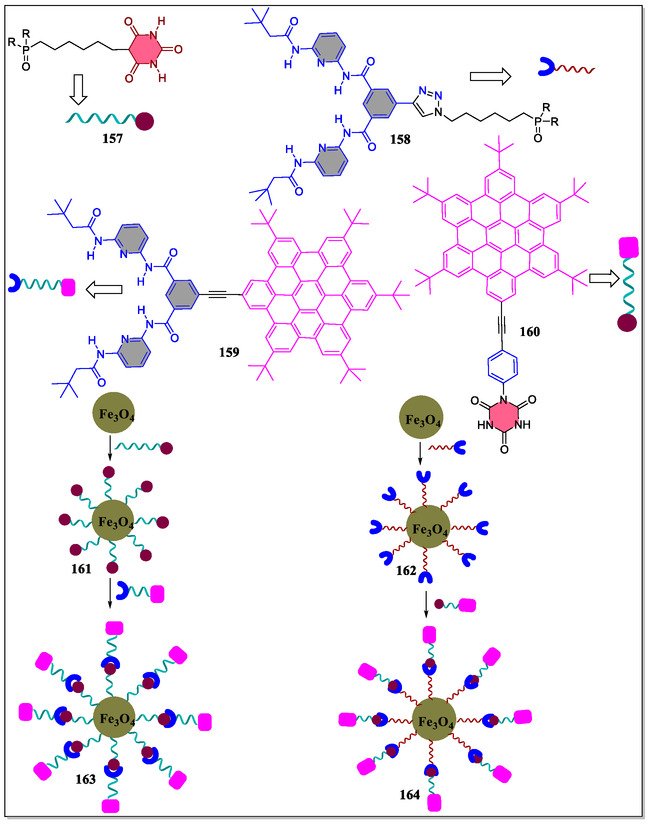
Structural analysis of superparamagnetic iron oxide nanoparticles modified with Hamilton receptor and cyanurate phosphoric acid.

Hirsch's group has developed new superparamagnetic iron oxide nanoparticles with cyanurate derivative and Hamilton receptors for hydrogen bonding interactions (**Figure** **33**). The study investigated molecular and surface interactions by adjusting receptor and guest molecule positions. The second nanoparticle (**161** and **162**) had more double molecules due to small cyanurate moieties. Surface hydrogen bonding interactions were three times stronger than in solution. Hamilton receptor placement had minimal effects, while bulky hexabenzocoronene impacted molecule coordination. Binding of **160** with **163** was hindered, while **159** with **161** was stabilized. Each receptor molecule on the surface could bind multiple guest molecules. These findings advance supramolecular complexation in hybrid organic–inorganic systems for potential applications in drug delivery, nanosensing, and nanomaterial devices.^[^ ^164^ ^]^

## 7 PET Properties Displayed by Hamilton‐Type Systems

**Figure 34:**
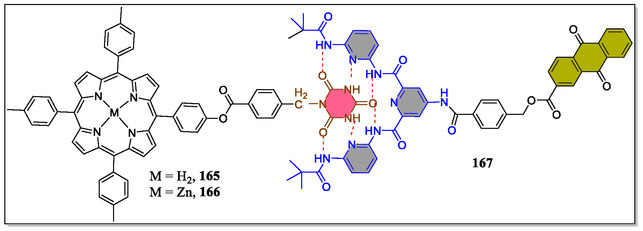
Complexation of receptor (**167**) with porphyrin‐based barbiturate (**165**, **166**).

Hamilton receptor display strong binding with cyanuric and barbituric acids with potential directionality, and serves as an excellent system for illustrating the principles of electron transfer process.^[^ ^52^, ^69^ ^]^ On the contrary, porphyrins with metal complexes are considered as a class of molecules, familiar in displaying potential spectroscopic, photochemical, and electrochemical properties. The PET between quinone and porphyrin derivatives, biomimetic the initial process of photosynthetic systems, thereby attaining significant attention from scientific community.^[^ ^165^, ^166^ ^]^ In this regard, Zhao et al. have synthesized and reported the photophysical properties of noncovalent systems containing porphyrin‐**165**, zinc‐porphyrin‐cyanuric acid **166** conjugates, and anthraquinone attached with Hamilton receptor **167** (**Figure** **34**).^[^ ^167^ ^]^ From the fluorescence titration experiments, the binding constants of both the supramolecular moieties were determined of about (2.8 ± 0.3) × 10^3^ mol^−1^ L. The singlet electron transfer arises between the donor and acceptor within the supramolecular assemblies having rate constants and efficiencies 7.6 × 10^7^ and 43%, respectively for **165**/**167**, and 7.0 × 10^8^ s^−1^ and 58% for **166**/**167**. The intramolecular electron transfer proceeds mainly by means of ‘*through space*’ mechanism. From the observation of physical properties, it was revealed that the Hamilton‐based H‐bonding systems are promising candidate for electron transfer processes in the biological systems.

**Figure 35:**
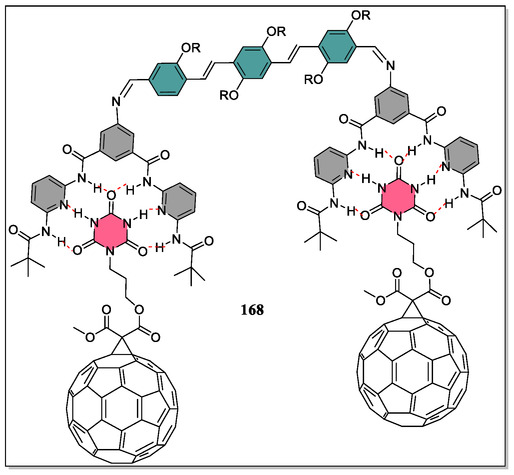
Complexation structure of OPV‐based Hamilton receptor and C_60_‐based barbiturate.

In contrast, Pagona et al. have reported the H‐bonding association between C_60_‐barbiturate and oligophenylenevinylene (OPV)‐based HR (**Figure** **35**).^[^ ^77^ ^]^ The formation of stable complex having association constant (10^5^ M^−1^) within 1:2 stoichiometric suggests the high stability between OPV and C_60_ (Figure 35). The time‐resolved spectroscopic and steady‐state analysis revealed rapid PET to C_60_ during excitation of OPV unit, which results in the charge separation within the complex **168** with *k*_*CS*_ 4.1 × 10^11^ s^−1^. The intracomplex collisions of OPV and C_60_ fragments are avoided, and hence, suggesting that the pattern of six H‐bonding per OPV terminal produces the PET. These complex super structures are promising material for PET phenomena and have high importance for host–guest complexes as well as for the construction of novel optoelectronics and solar cell materials.

**Figure 36:**
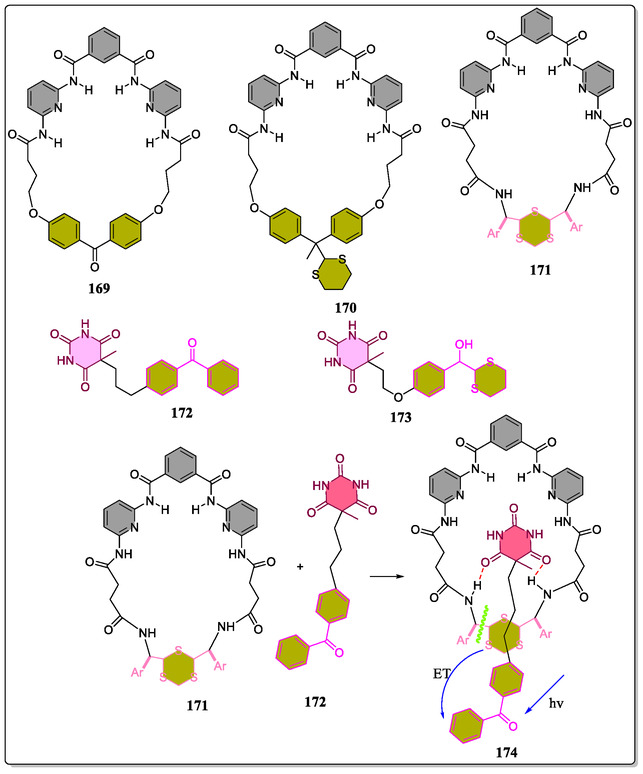
Structural representation of novel photoactive receptors.

Kutateladze and coworkers have designed and synthesized novel photoactive receptors for the barbiturate, which display lock and key mechanism upon external sensitizer (**Figure** **36**).^[^ ^168^ ^]^ It could inspected from the Figure 36 that the receptors **169–171** having benzophenone (BP) moiety, dithiane adduct, and photolabile trithiane, forms the macroheterocycle. Photolysis was carried out at the wavelength ranged 350–360 nm, stated by the sensitizers absorption and photoinduced fragmentation in macrocycle **172** illustrating the lock and key system, where irradiation of “key” **173** causes fragmentation of macrocycle thereby unlocking the lock. In this article, below 0.5–1 mM concentrations, the free BP does not sensitize fragmentation in **173**; however, sensitizer **172** outfitted with barbiturate. The unlocking process is accompanied by the release of barbiturate, monitored by the NMR spectroscopy. On the contrary, the conditional release of dithiane occurs from **171** or **172**. The photoinduced fragmentations of binary systems are possible when molecular recognition event arms the system, thereby making it light‐sensitive. These types of systems may be potentially beneficial for bioanalytical applications, in which molecular recognition process is recognized by photoinduced dithiane release in bulk solution.

**Figure 37:**
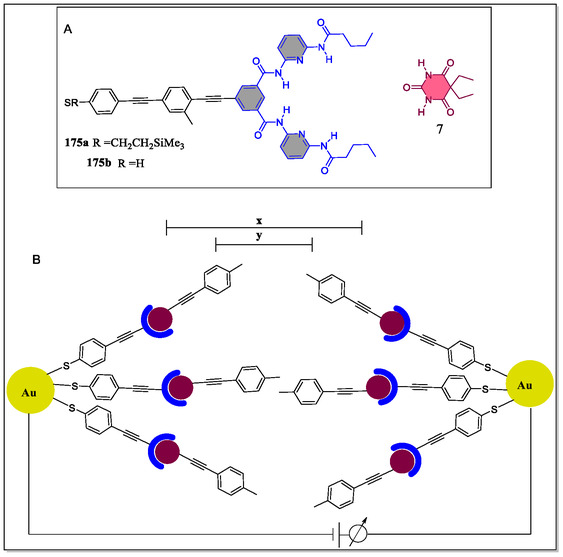
A) Chemical structures of HR and the diethyl barbiturate; B) diagrammatic view of a single nanowire gap in conductivity measurement setup.

In a separate article, Lüning and coworkers have reported the synthesis of a fully conjugated Hamilton‐based receptor, displaying H‐bonding with guest molecule on one side, whereas the other end have sulphur unit for immobilization of gold clusters (**Figure** **37**).^[^ ^169^ ^]^ The binding and adsorption of Hamilton receptor on the gold was examined by the fluorescence spectroscopy revealing the decrease in fluorescence upon mixing of **175a** with gold clusters, thereby confirming that **175b** is physically adsorbed onto the gold nanoclusters and desorbs upon dilution. The gold particles attached on nanowire gap find application in conductivity measurements, from the inspection of Figure 36(**B**), the distance **x** is large, which is present when the conducting host material is attached with the gold clusters, whereas, the distance **y** is low, which is present when the conducting guests systems are bound. On the contrary, from the studies of ^1^H‐NMR titration, it was confirmed that **175** displayed high binding affinities toward diethylbarbiturate **7** with association constant of about 40 000 M^−1^ in chloroform solvent at 298 K.

**Figure 38:**
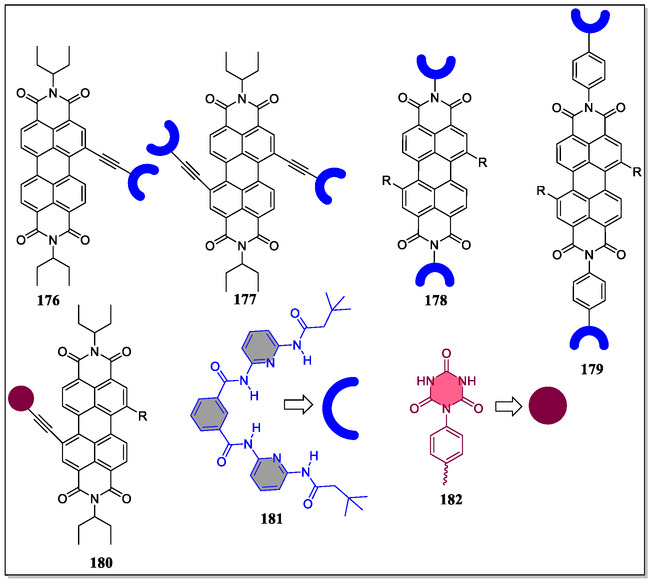
Chemical structures of Hamilton receptor based PDI molecules and cyanuric acid based PDI molecule.

The research team of Berlin have reported a high yielding approach for the inclusion of HR onto the bay and imide region of the perylenediimide (PDI) (**Figure** **38**).^[^ ^170^ ^]^ Two different Hamilton‐based PDI molecules in which isophthalamide unit of Hamilton receptor is introduced directly, and other one is attached through a phenylacetylene group (conjugated spacer) to the PDI. The comparative studies of these two receptors reveals a slight change in bathochromic shift due to extended conjugation, whereas, alike pattern of three reductions were observed in both the cases. Instead, a considerable difference was observed while comparing different substituents of isophthalamide component 1‐ or both 1 and 7‐positions of the PDI. However, the photophysical properties of all the derivatives of PDI did not display any dramatic change. From the analysis, it was observed that the presence of various reduction peaks in these types of Hamilton‐based PDI molecules makes them interesting and promising candidates for the development of future organic electronic devices.

## 8 Supramolecular Catalysis

**Figure 39:**
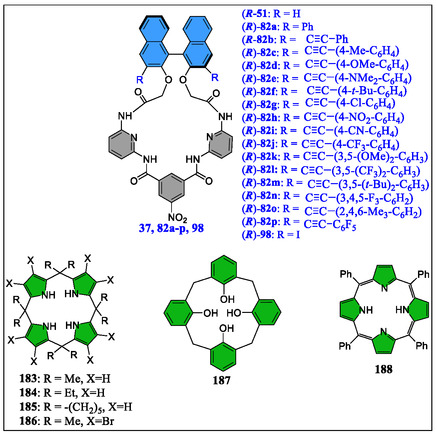
Chemical structures of some vital macrocyclic catalysts (**183–186**), **37**, **82a‐p**, and **98** used for the preparation of cyclic carbonates.

Gratifyingly, cyclic carbonates finds promising applications in lithium‐ion secondary batteries as electrolytes, and also utilized as raw materials for the synthesis of polyurethanes or polycarbonates. These types of compounds display an important role as the intermediate compounds leading to generate value‐added molecules. In order to quantify the yields as well as to reduce the toxic byproducts, several catalytic synthetic approaches have been optimized for the construction of cyclic carbonates.^[^ ^171^, ^172^, ^173^, ^174^, ^175^, ^176^, ^177^, ^178^, ^179^, ^180^, ^181^, ^182^ ^]^ To this regard, in recent years, various macrocyclic catalysts have been optimized to explore the preparation of cyclic carbonates utilizing CO_2_ and epoxides (**Figure** **39**).^[^ ^183^, ^184^, ^185^, ^186^, ^187^, ^188^, ^189^, ^190^, ^191^, ^192^ ^]^

**Scheme 1:**
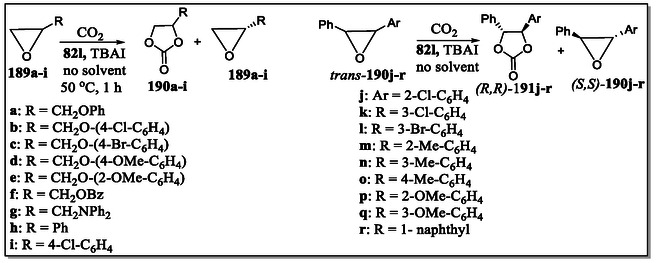
Synthesis of cyclic carbonates from epoxides and CO_2_ using the Hamilton receptor **82i** as organocatalyst.

**Scheme 2:**
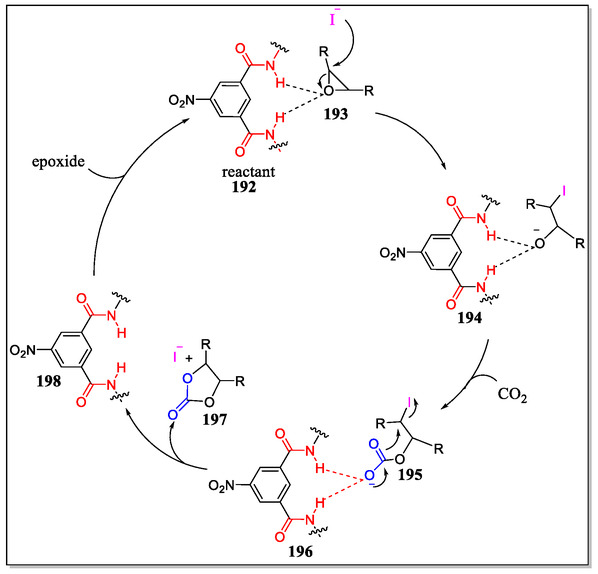
Mechanistic approach of Hamilton catalyst toward epoxides and alcohols.

Ema et al. have reported the synthesis of chiral macrocyclic organocatlyst **37**, **82a‐p**, and **98** for the preparation of cyclic carbonates from CO_2_ and epoxides (**Scheme** **1**). Due to the presence of chiral BINOL scaffold and H‐bonding active sites, enantioselective activation of epoxides was realized by these authors. The proposed mechanism for the catalytic conversions of epoxides and CO_2_ into cyclic carbonates is shown in the **Scheme** **2**. From the results of various synthesized macrocyclic catalysts, it was revealed that the catalytic system **82l** was considered as most promising organocatalyst for the enantioselective synthesis of cyclic carbonates from mono‐ and disubstituted epoxides and CO_2_.^[^ ^193^, ^194^ ^]^

## 9 Conclusions and Concluding Remarks

**Figure 40:**
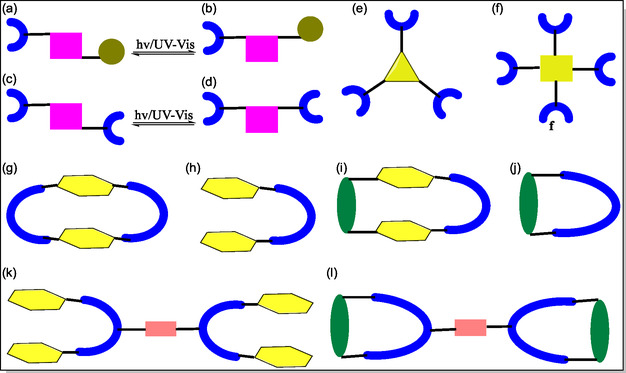
a–i) Pictorial structural representations displaying various types of possible functionalization of Hamilton receptor, most of these proposed structures are not yet synthesized.

This review article describes importance of Hamilton receptor in molecular recognition of various barbital types molecules, moreover which includes also anions, uric acid, and drug molecules. On the contrary, its derivatives preferably display catalysis and photo‐electronic properties which were elaborated in detail. Due to its convenient and valuable functionalization, the Hamilton‐type receptor display yet much more intersecting applications in the domain of polymeric materials, molecular devices, artificial molecular machines, and biomedical, and bioanalytical as well as in pharmaceutical science. Although, various advantageous and potentially fruitful results have been obtained from the functionalized HR in the area of chemical, physical, biological, supramolecular, and medicinal chemistry, but we believe that the parent HR is not yet fully explored and the best is yet to come. Since, the parent Hamilton receptor has various available active positions for further possible functionalization to give myriad of functionalized HR. Here, we have listed some ways for the functionalization of Hamilton receptor, among which some have been synthesized and explored (**Figure** **40**a–i). But as per our best knowledge that most of the functionalized structures are yet to be explored in future. However, the synthesis of these types of Hamilton‐type receptors may be challenging, tedious, and difficult due its solubility issues. Interestingly, these types of compounds could afford new opportunities beyond the horizon of supramolecular chemistry throughout the world. We believe that this manuscript will present timely and valuable reference for researcher, interested on Hamilton‐type receptors in particular and supramolecular chemistry in general as well as inspire them from academic to industrial level for the construction and studies of novel molecular receptors including novel Hamilton‐type receptors.

## Conflict of Interest

The authors declare no conflict of interest.
